# Spatial pattern of cell geometry and cell-division orientation in zebrafish lens epithelium

**DOI:** 10.1242/bio.20149563

**Published:** 2014-09-26

**Authors:** Toshiaki Mochizuki, Shohei Suzuki, Ichiro Masai

**Affiliations:** Developmental Neurobiology Unit, Okinawa Institute of Science and Technology Graduate University, 1919-1 Tancha, Onna, Okinawa 904-0495, Japan

**Keywords:** zebrafish, lens, cell division, cell proliferation, E-cadherin

## Abstract

Cell proliferation is a key regulator of tissue morphogenesis. We examined cell proliferation and cell division in zebrafish lens epithelium by visualizing cell-cycle phases and nuclear positions, using fluorescent-labeled geminin and histone proteins. Proliferation was low in the anterior region of lens epithelium and higher in the marginal zone anterior to the equator, suggesting that the proliferation zone, called the germinative zone, is formed in zebrafish lens. Interestingly, cell-division orientation was biased longitudinally in the anterior region, shifted from longitudinal to circumferential along the anterior–posterior axis of lens sphere, and was biased circumferentially in the peripheral region. These data suggest that cell-division orientation is spatially regulated in zebrafish lens epithelium. The Hertwig rule indicates that cells tend to divide along their long axes. Orientation of long axes and cell division were biased similarly in zebrafish lens epithelium, suggesting that cell geometry correlates with cell-division orientation. A cell adhesion molecule, E-cadherin, is expressed in lens epithelium. In a zebrafish *e-cadherin* mutant, the long axes and cell-division orientation were shifted more longitudinally. These data suggest that E-cadherin is required for the spatial pattern of cell geometry and cell-division orientation in zebrafish lens epithelium.

## INTRODUCTION

Cell proliferation, especially cell-division orientation, is a key regulator in the organization and shaping of tissues (Gillies and Cabernard, 2011). Over 120 years ago, Oscar Hertwig discovered that cells divide along their long axes, which is known as the “long-axis rule”, suggesting that cell shape is a determinant of spindle and cell-division orientation (Hertwig, 1884). *In vitro* studies using cultured human cells supported this rule, and demonstrated that cell-division orientation and mitotic spindle positioning depend on physical environmental parameters such as cell shape (Gibson et al., 2011; Minc et al., 2011), extrinsic force (Fink et al., 2011), and cell-substrate adhesion (Théry et al., 2007; Théry et al., 2005; Toyoshima and Nishida, 2007). In addition to these physical parameters, *in vivo* studies using animal models have revealed extrinsic and intrinsic cellular mechanisms that determine spindle orientation during cell division (Morin and Bellaïche, 2011). In *Drosophila*, proto-cadherin molecules, Fat and Dachsous, regulate planar polarization of the atypical myosin, Dachs, to alter the apical geometry of cell shape, which subsequently determines the orientation of the mitotic spindle (Mao et al., 2011). During zebrafish gastrulation (Concha and Adams, 1998), cell division is oriented contrary to the long axis and is regulated by a non-canonical Wnt pathway, which may override physical parameter-mediated spindle positioning (Gong et al., 2004).

The lens is a unique system for studying epithelial proliferation and morphogenesis. It is composed of epithelial cells, which form a monolayer on the anterior surface of the tissue, and fiber cells, which constitute the remainder and the majority of the tissue volume (Bassnett et al., 1999; Dahm et al., 2007; Greiling and Clark, 2009; Lovicu and McAvoy, 2005). The peripheral margin of lens epithelium is called the equator, where lens epithelial cells start to differentiate into fiber cells. Differentiating lens fiber cells elongate along their antero-posterior (AP) axes and cover the old lens fiber cores. In late stages, nuclei and other cytoplasmic organelles are degraded to minimize light scattering (Bassnett, 1995; Bassnett and Beebe, 1992; Bassnett and Mataic, 1997). Classic studies on chick, rat, and mouse lenses revealed that cell proliferation is active in the peripheral zone of lens epithelium, called the “germinative zone”, which is positioned anterior to the equator. In contrast, cell proliferation is low in the anterior-most region (McAvoy, 1978; Modak et al., 1968; Zhou et al., 2006). Although several extrinsic and intrinsic molecules regulate switching from cell proliferation to differentiation through the equator (Mochizuki and Masai, 2014), molecular mechanisms that spatially coordinate cell proliferation in the lens epithelium remain to be elucidated.

In this study, we examined cell proliferation and cell division in zebrafish lens epithelium, using a zebrafish transgenic line expressing fluorescently labeled Geminin and Histone2A, which visualize cell-cycle phases. Cell proliferation was active in the peripheral region anterior to the equator, suggesting that the germinative zone is formed in zebrafish. Furthermore, cell-division orientation was biased longitudinally in the anterior region, shifted from longitudinal to circumferential along the AP axis, and was biased circumferentially in the peripheral region, suggesting that cell-division orientation is spatially regulated in lens epithelium. Next, we examined mechanisms that regulate cell-division orientation. The Hertwig rule indicates that cells tend to divide along their long axes. In zebrafish, the long axis of lens epithelial cells was biased longitudinally in the anterior region and circumferentially in the peripheral region, suggesting that cell-division orientation correlates with apical geometry. A cell adhesion molecule, E-cadherin (cadherin1, cdh1) is expressed in the zebrafish lens epithelium. In a zebrafish *e-cadherin* mutant, orientation of the long axis and cell division were shifted more longitudinally. These data suggest that E-cadherin is required for the spatial pattern of cell-division orientation and cell apical geometry in zebrafish lens epithelium.

## MATERIALS AND METHODS

### Fish

Zebrafish (*Danio rerio*) were maintained at 28.5°C according to a standard protocol (Westerfield, 1993). Riken wild type, OIST wild type, and WIK were used to establish transgenic fish strains. Two zebrafish transgenic lines, *Tg(h2afv:GFP)*^kca6/kca66^ (Pauls et al., 2001) and *Tg(EF1α: mCherry-zGem)*^oki011^, were used. *half baked* (*hab*)^rk3^ (Shimizu et al., 2005) was used as an *e-cadherin*/*cdh1* mutant. Protocol for animal care and experiment has been approved by OIST Institutional Animal Care and Use Committee.

### Generation of the zebrafish transgenic line, *Tg(EF1α: mCherry-zGem)*

Dr. Atsushi Miyawaki (RIKEN BSI) provided the Tol2 transposon-based expression plasmid, pT2KXIGD, which expresses an mCherry-fused, 100-amino acid peptide of zebrafish Geminin (mCherry-zGem) (Genbank accession number: NM_200086) under the control of the *EF1α* promoter. This plasmid was injected into zebrafish eggs at the one-cell stage with *in vitro*-synthesized transposase mRNA (Urasaki et al., 2006). After injection, mCherry fluorescence-positive embryos were selected at 24 hr post-fertilization (hpf) and bred to establish a zebrafish transgenic line, *Tg(EF1α: mCherry-zGem).* We generated four independent transgenic lines, and used one allele, *Tg(EF1α: mCherry-zGem)*^oki011^, for this study.

### Time-lapse scanning of zebrafish lens epithelium

The *Tg(h2afv:GFP)* transgenic strain expresses a zebrafish histone2A variant, His2AvD, fused with GFP at the C-terminus, under control of the endogenous *His2AvD* promoter (Pauls et al., 2001). We crossed two transgenic fish, *Tg(h2afv:GFP)* and *Tg(EF1α:mCherry-zGem)* and generated double transgenic embryos. These were scanned at 24°C using an upright Zeiss LSM710 confocal laser scanning microscope with water-immersion objective lens. For confocal scanning of lens epithelium, left lenses were used. Lens epithelium of wild-type embryos was scanned along the AP axis every 15 minutes for 8–12 hr in three time windows: 33–45, 49–61, and 62–72 hpf (supplementary material Fig. S1). Lens epithelium of *hab* mutant embryos was similarly scanned for 12 hr: 33–45 hpf (supplementary material Fig. S2). To determine mCherry-zGem expression levels quantitatively (supplementary material Fig. S3C), the intensity of mCherry fluorescence was measured with Image J software (NIH, USA).

### Determination of cell-division orientation

We acquired time-lapse 3D images of lens epithelium, each of which consists of sections along the AP axis of the lens. Positions of individual lens epithelial cells were defined by their nuclear positions, which were visualized with *Tg(h2afv:GFP)* fluorescence.

Supplementary material Fig. S4 illustrates the procedure to calculate cell-division orientation of A cell, which generates two daughter cells, the A′ cell and A″ cell (supplementary material Fig. S4A). On the projection image along the AP axis (supplementary material Fig. S4B), we measured cell-division orientation as τ, the angle between the line that connects projected positions of two daughter cell nuclei, A_pro_′ and A_pro_″, and the tangential line that crosses mother nucleus along the lens circumference (blue line in supplementary material Fig. S4B).

Next, we estimated cell-division orientation on the real lens epithelium, τ′, which is defined as the angle between the line that connects two daughter cell nuclei (A′–A″) and the circumferential line of the lens sphere that crosses the mother nucleus (red line in supplementary material Fig. S4A). To calculate τ′, we focused on the tangential plane that contacts both the longitudinal and circumferential lines that cross the mother nucleus (supplementary material Fig. S4C). For this calculation, we selected two arbitrary cells, B and C, that were located most closely adjacent to the mother cell along the longitudinal line, and measured r_b_ and r_c_, the radius of lens plane that contains B and C cells, respectively, and z_b_ and z_c_, z-position (distance from the most anterior top) of the lens plane that contains the B and C cells, respectively. θ was defined as the angle between the horizontal plane and the tangential line that crosses the mother nucleus of the A cell along the longitudinal line of the lens sphere (supplementary material Fig. S4C). If the tangential line that crosses the mother nucleus of the A cell along the longitudinal line was approximated as the line that connects the B and C cell nuclei, θ is measured by the equation: tanθ = (z_c_ − z_b_)/(r_c_ − r_b_) (Eqn 1) (supplementary material Fig. S4D), τ′ is calculated by the equation: tanτ′ = tanτ/cosθ (Eqn 2) (supplementary material Fig. S5). We determined cell-division orientation of all mitotic positions for 12 hr using three sets of scanning data for each of the three time windows of wild-type and one time window of *hab* mutant.

### Measurement of the long-axis orientation, perimeter length, and vertex number

Zebrafish gastrula-stage embryos were labeled with a fluorescent lipid dye, Bodipy ceramide (Molecular Probes, B22650), as previously described (Masai et al., 2003). Left lenses were scanned using the confocal microscope. In order to scan the peripheral region after 58 hpf, lenses were dissected from the eyes and mounted in Ringer's solution. A vertex was defined as the point where three cells meet, and the number of vertices was counted for individual cells. The long (maximal) and short (minimal) axes were defined using the “feret diameter measurement” of Image J software (NIH). The long-axis orientation was defined as the angle between the long axis and the circumferential line of the lens sphere.

### Normalization of lens size differences and subdivision of lens epithelium of wild-type and *hab* mutants

To compare cell division orientation more precisely between wild type and *hab*^rk3^ mutants, we divided the lens epithelium into three regions: anterior, intermediate, and peripheral. Although lens epithelial cell proliferation and lens fiber cell differentiation proceeded normally in *hab*^rk3^ mutants, the average size of the lens sphere was slightly smaller in *hab*^rk3^ mutants. The equatorial radius of three *hab*^rk3^ mutant lenses (supplementary material Fig. S2: r = 31, 33, 36 µm: in average 33 µm) was slightly smaller than in those of wild-type lenses (supplementary material Fig. S1: r = 32, 36, 37 µm; in average 35 µm). Consistently, the mitotic peak occurred in the peripheral region (r = 26–30 µm) in *hab*^rk3^ mutant lenses, whereas it was r = 31–35 µm in wild type lenses (see Results, e.g. Fig. 6B). Thus, we defined r = 26–40 µm and r = 21–35 µm as the peripheral region, which corresponds to the proliferative, germinative zone, in wild type and *hab*^rk3^ mutants, respectively. In a radial section of the lens sphere, the peripheral region averages three lens epithelial cells along AP axis in wild type and *hab*^rk3^ mutants. Since the remaining lens epithelium from the anterior pole to the anterior edge of the peripheral region contained six lens epithelial cells in wild type and four cells in *hab*^rk3^ mutants along the AP axis, the remaining lens epithelium was divided into anterior and intermediate regions so that each region contained an equal number of lens epithelial cells. In this case, anterior and intermediate regions corresponded to r = 0–15, 16–25 µm for wild type and r = 0–10, 11–20 µm for *hab*^rk3^ mutants (see Results, e.g. Fig. 6D).

### BrdU labeling

Embryos were soaked with Ringer's solution containing 15% DMSO and 10 mM 5-Bromo-2′-deoxyuridine (BrdU) (Sigma, B5002) at 4°C for 10 min, and washed with water. After incubation at 28.5°C for 30 min, BrdU-incorporated embryos were fixed with 4% Paraformaldehyde (PFA). BrdU incorporation was detected as described previously (Imai et al., 2010).

### Histology

Antibody labeling of cryosections and *in situ* hybridization were carried out as described previously (Imai et al., 2010). Information about antibodies is shown in supplementary material Table S1.

### Labeling with Bodipy ceramide and mCherry-tjp1a

Full-length zebrafish tjp1a (ZO1) cDNA (Genbank no. XM_005168933) was amplified with polymerase chain reaction (PCR) using specific primers, and subcloned using the pCS2 expression vector. Synthesized tjp1a RNA (500 µg/mL) was injected into one-cell stage eggs, followed by Bodipy ceramide incorporation at the tail-bud stage. Procedures for Bodipy ceramide incorporation were carried out as previously described (Masai et al., 2003).

### Number of samples and statistical analyses

The number of lenses and lens epithelial cells examined in each experiment, and p-values are provided in supplementary material Fig. S6. For statistical analyses, n was equal to the number of lens epithelial cells, not the number of lenses.

## RESULTS

### Visualization of cell-cycle phases using zebrafish transgenic line *Tg(h2afv:GFP; EF1α:mCherry-zGem)*

**Fig. 1.**
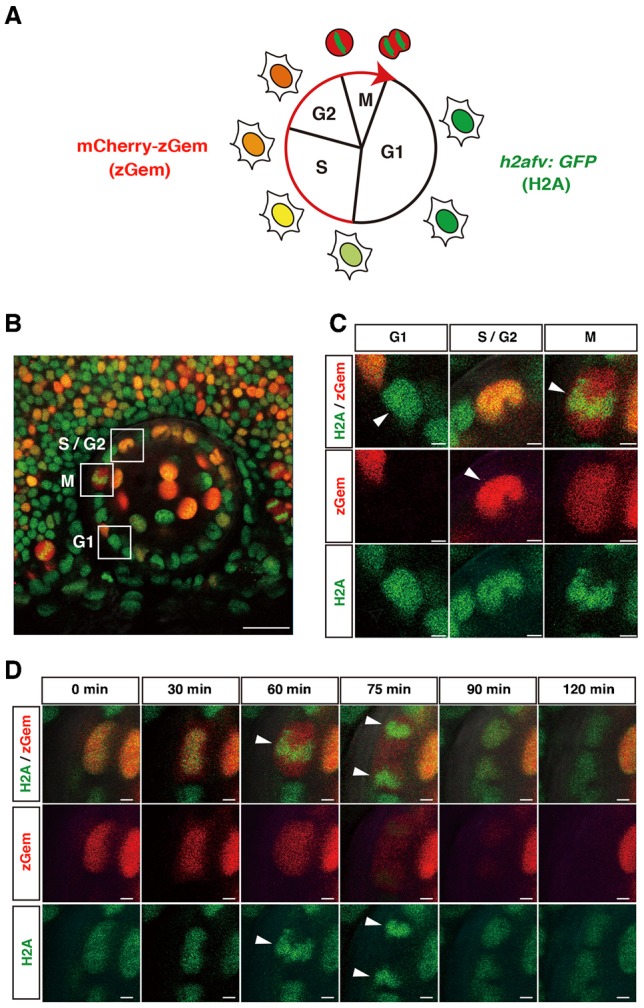
Visualization of cell-cycle phases in zebrafish transgenic line *Tg(h2afv:GFP; EF1α:mCherry-zGem)*. (A) Fluorescent expression in a zebrafish transgenic line *Tg(h2afv:GFP; EF1α:mCherry-zGem)*. In G1 phase, only GFP is expressed in the nucleus. mCherry fluorescence gradually increases during S phase and peaks in G2/M phase. In M phase, GFP fluorescence indicates condensed chromatin. (B) Lateral view of a 37 hpf lens of a zebrafish transgenic embryo *Tg(h2afv:GFP; EF1α:mCherry-zGem)*. Squares indicate cells in G1, S/G2 and M phases. (C) Higher magnification of squares shown in (B). Red and green channels are shown below. Arrowheads indicate GFP fluorescence in G1 phase, mCherry fluorescence in S/G2 phase, and condensed GFP fluorescence in M phase. (D) GFP and mCherry expression during M phase. During mitosis, GFP fluorescence is condensed and aligned along the plane of division (arrowhead in 60 min). GFP fluorescence is segregated in opposing directions (arrowheads at 75 min) and becomes a round shape at 90 min. mCherry fluorescence disappears at 120 min. Scale bars: 20 µm (B), 2 µm (C,D).

The fluorescent, ubiquitination-based cell-cycle indicator (Fucci) was reported as a fluorescent system that visualizes G1 and S/G2/M phases with fluorescent protein-tagged peptides of the cell cycle regulators, Cdt1 and Geminin, respectively (Sakaue-Sawano et al., 2008). In the zebrafish version of Fucci, which is called cell-cycle illuminated (Cecyil), the monomeric Azami Green-tagged zebrafish Geminin N-terminal domain (100 amino acids) (mAG-zGem) and the monomeric Kusabira Orange2-tagged zebrafish Cdt1 N-terminal domain (190 amino acids) (mKO2-zCdt1) enable us to distinguish two different cell-cycle phases, S/G2/M and G1, respectively (Sugiyama et al., 2009). Although the original Cecyil transgenic line is powerful for live imaging of cell-cycle progression, fluorescent detection is less efficient in highly proliferating tissues such as the retina. mAG-zGem fluorescence in S/G2/M phases becomes weak in zebrafish retina after 48 hpf, because transcription by the *EF1α* promoter declines in later stages. Furthermore, mKO2-zCdt1 fluorescence in G1 phase becomes also weak in zebrafish retina after 30 hpf, because the *EF1α* promoter does not drive enough transcription due to a very short G1 phase. To improve fluorescence intensity in S/G2/M phases, we replaced mAG with mCherry to generate transgenic zebrafish expressing mCherry-zGem under control of the *EF1α* promoter, namely *Tg(EF1α: mCherry-zGem)* (Fig. 1A). To monitor G1 phase, we used another zebrafish transgenic line *Tg(h2afv:GFP)* instead of *Tg(mKO2-zCdt1)* (Fig. 1A). The *Tg(h2afv:GFP*) transgenic line expresses zebrafish H2avD, a variant of Histone2A, fused with GFP at the C-terminus, and visualizes nuclear-associated chromatin (Gong et al., 2004; Pauls et al., 2001).

We generated a double transgenic fish line, *Tg(h2afv:GFP; EF1α:mCherry-zGem)*, and scanned GFP and mCherry fluorescence in the neural retina and in the lens epithelium at 37, 50, and 63 hpf (supplementary material Fig. S3A and Movies 1–3 for 3D image at 37, 50, and 63 hpf). In both tissues, three cell types were distinguishable (Fig. 1B,C). The first cell type expressed only GFP, indicating G1 phase. The second cell type expressed both GFP and mCherry, indicating S/G2 phase. The expression level of mCherry was weak at the beginning of S phase, but gradually increased to reach a plateau at the end of S phase, and then declined in M phase (supplementary material Fig. S3B,C and Movie 4). The third cell type expressed a high level of mCherry with condensed GFP. Time-lapse scanning revealed that this condensed GFP expression represents chromosomal condensation during mitosis (Fig. 1D; supplementary material Movie 5). During M phase, GFP was initially observed in the whole nucleus, accumulated centrally and condensed in prophase, aligned along the equatorial plane in metaphase, and divided in two sets in anaphase, which were inherited by daughter cells. This profile suggests that the third cell type comprises M-phase cells. Labeling of *Tg(h2afv:GFP; EF1α:mCherry-zGem)* lens with BrdU and anti-phosphorylated histone H3 (pH3) antibody confirmed that mCherry-positive cells contain cells undergoing S and M phases (supplementary material Fig. S7). These observations suggest that three cell-cycle phases, G1, S/G2, and M, are distinguishable, using *Tg(h2afv:GFP; EF1α:mCherry-zGem)*.

### Cell proliferation is active in the peripheral region of lens epithelium

**Fig. 2.**
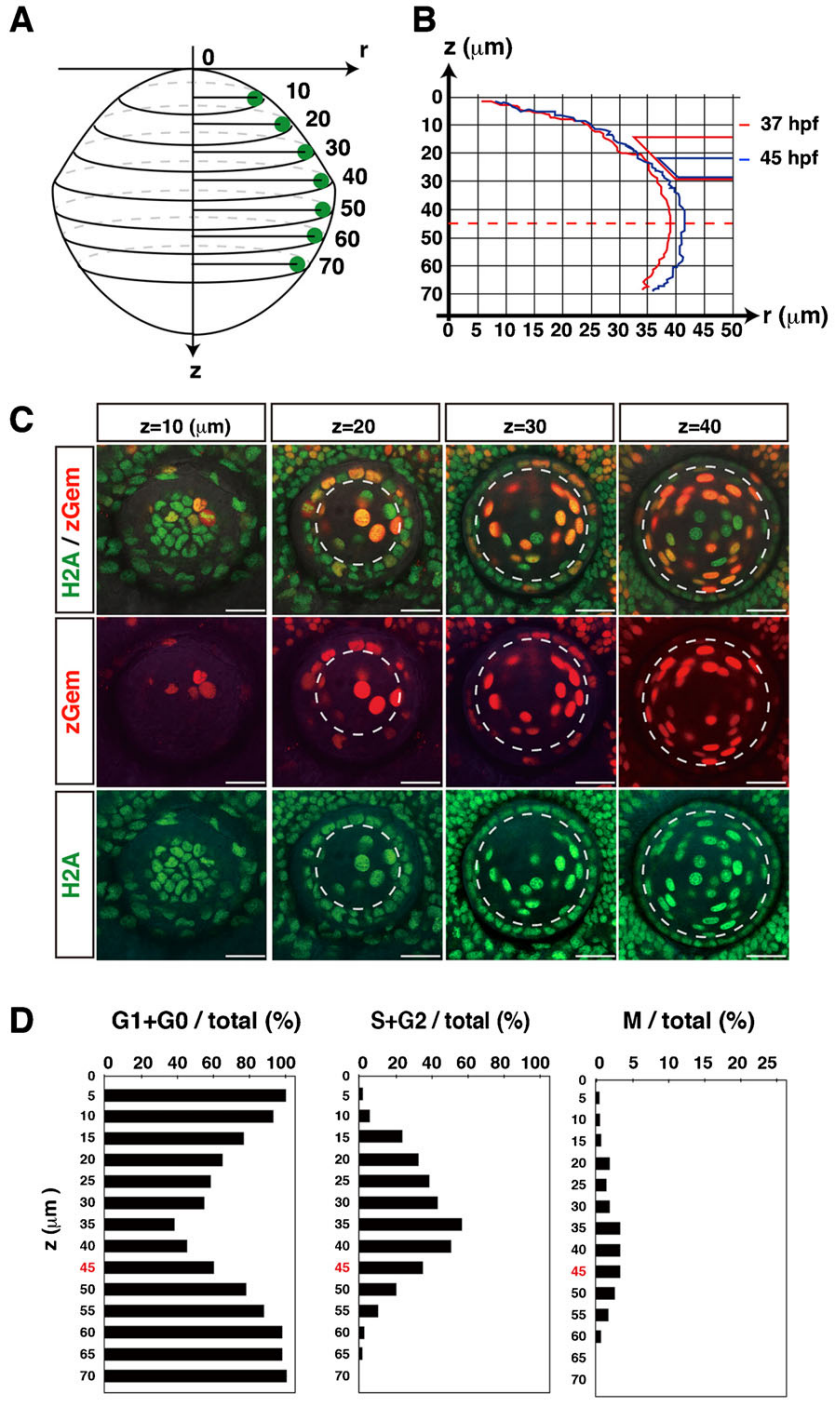
Spatial cell-cycle pattern in zebrafish lens epithelium. (A) Coordinates of the lens. The “z-axis” indicates the distance from the antero-most plane. The “r-axis” indicates the radius in the plane perpendicular to the z-axis. (B) Nuclear positions of lens epithelial cells for a particular lens at the beginning (37 hpf, red line) and the end (45 hpf, blue line) of the scanned period. Right red and blue trapezia indicate the retinal ciliary marginal zone that was associated with the lens surface at 37 and 45 hpf, respectively. During the period from 37 to 45 hpf, the lens grew to be slightly larger, but the z-position of the equator was not changed at z = 45 µm. (C) Confocal images of the lens along the z-axis at 37 hpf. The lens epithelium is distinguished as monolayer covering the lens fiber core. Dotted lines indicate the boundary between the lens epithelium and the lens fiber core. (D) Averaged percentages of G1/G0, S/G2 and M phase cells relative to the total number of lens epithelial cells along the z-axis, calculated using time-lapse scanning images every 15 min during the period from 37 to 45 hpf. The equator position z = 45 µm is indicated by red color. Scale bars: 20 µm.

Previous studies have revealed that DNA synthesis is active in the peripheral margin of lens epithelium anterior to the equator in rat, mouse, and chick (McAvoy, 1978; Modak et al., 1968; Zhou et al., 2006). This peripheral proliferating zone is called the germinative zone. To determine whether zebrafish lens epithelium has a similar proliferation zone, we examined GFP and mCherry fluorescence using the zebrafish transgenic line *Tg(h2afv:GFP; EF1α:mCherry-zGem)*. Here we examined one wild-type lens and scanned confocal images along the AP axis for 8 hours from 37 to 45 hpf. The position on the AP axis was shown as a z value, which indicates the distance from the anterior-most plane along the AP axis (Fig. 2A). From 37 to 45 hpf, the lens grew larger, but the equatorial position, which we defined as a circumferential line at the long radius of the lens sphere, was not changed (z = 45 µm) in this case (Fig. 2B). We examined GFP and mCherry fluorescence and determined which lens epithelial cells were in G1/G0, S+G2, and M phases along the AP axis (Fig. 2C,D). At the most anterior region z = 0 µm, most lens epithelial cells were in G0/G1 phase (left histogram of Fig. 2D). The fraction of G0/G1 phase cells decreased in a posterior direction, reaching a minimum of 40% at z = 35 µm, and increased in the region posterior to z = 40 µm. Consistently, the fraction of S/G2 phase cells was nearly 0% at z = 0 µm, gradually increased to a maximum of 57% at z = 35 µm, and decreased to 35% at the equator (z = 45 µm), and 20% at z = 50 µm (middle panel of Fig. 2D). Similar to the fraction of S/G2 phase cells, the fraction of M phase cells was very low in the anterior-most plane, increased to a peak of 3% from z = 35–45 µm, which is just anterior to the equator, and decreased again in the region posterior to z = 50 µm (right panel of Fig. 2D). These data suggest that proliferation is low in anterior region, gradually increases in a posterior direction and is highest in the peripheral region anterior to the equator in the zebrafish lens epithelium. Taken together, these data suggest that a highly proliferating region corresponding to the germinative zone is formed in zebrafish lens.

**Fig. 3.**
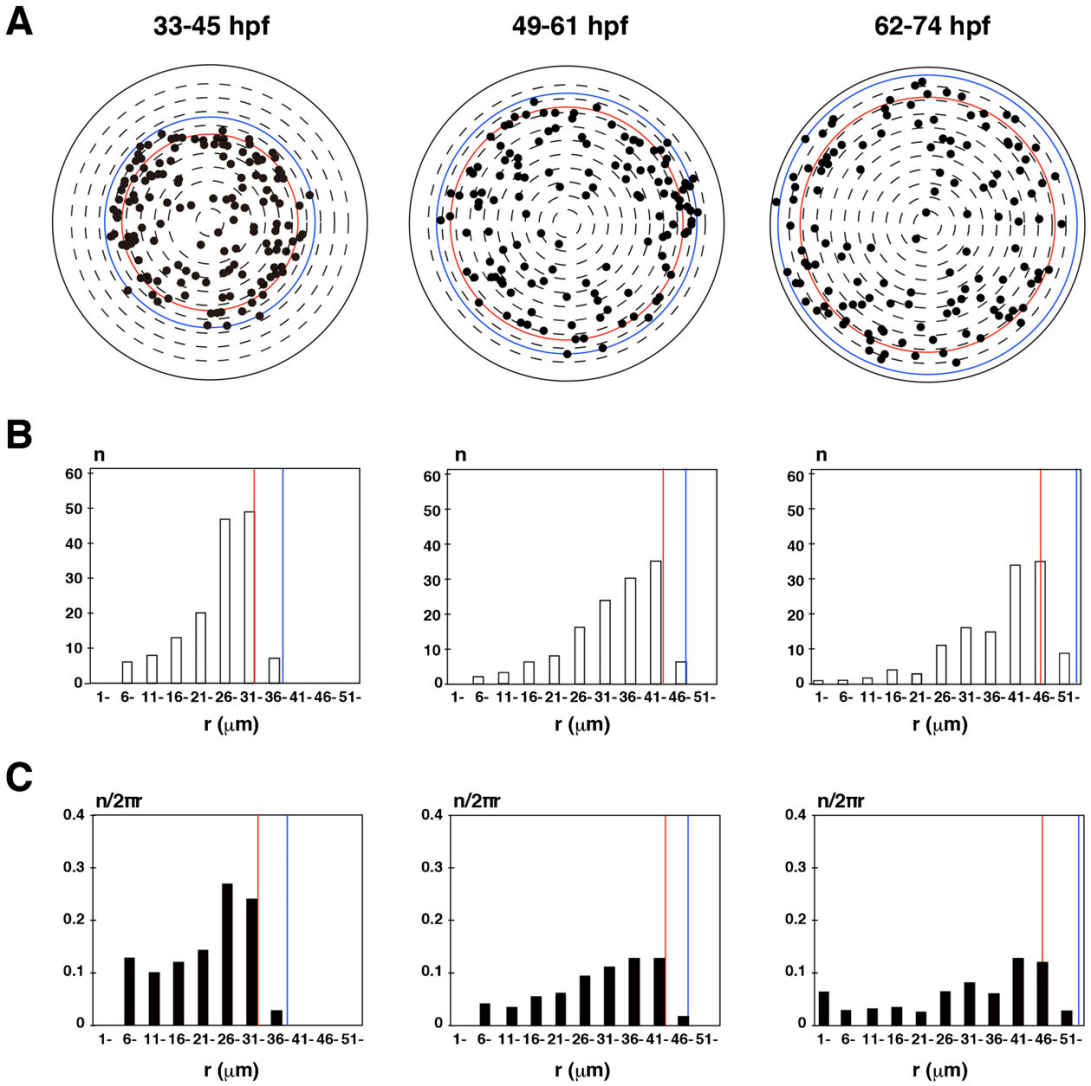
Position of mitotic cells in zebrafish lens epithelium. (A) Plotting of mitotic positions (black circle) in projection view of the lens epithelium in different time windows: 33–45, 49–61, 62–74 hpf. All data were merged from three lenses scanned in the same time windows. Dorsal is up and nasal is left. The density of mitoses is low in the anterior region and high in the peripheral region. The red line indicates the equatorial position of the smallest lens among three scanned lenses shown in supplementary material Fig. S1 at the beginning of the scanned period, whereas the blue line indicates the equatorial position of the largest lens among the same three lenses at the end of the scanned period. (B) Histogram of the number of mitotic cells along the r-axis. The number increases with r, reaching its highest level anterior to the equator, and then decreasing posteriorly. (C) Histogram of the density of mitotic cells along the r-axis (n/2nr). The peak of mitotic cell density is just anterior to the equator.

Next, we scanned GFP and mCherry fluorescence in the lens epithelium of zebrafish *Tg(h2afv:GFP; EF1α:mCherry-zGem)* transgenic embryos in a time-lapse manner with 12-hr time windows: 33–45 hpf, 49–61 hpf and 62–74 hpf (supplementary material Movie 6 for time-lapse images from 33–45 hpf). We examined spatio-temporal patterns of mitotic positions. In this analysis, we used three independent lenses for each time window (supplementary material Fig. S1A) and determined their equatorial positions during the scanned period (supplementary material Fig. S1B). Since most mitoses occurred in the region anterior to the equator, we plotted these mitotic positions in all three lenses on the AP-axis projection view for each of time windows (Fig. 3A). In all three time-windows, the number of mitoses was low anteriorly, increased toward the periphery, reached its zenith in the peripheral region anterior to the equator, and rapidly declined in the equatorial zone (Fig. 3B). Mitotic density showed a similar spatial profile (Fig. 3C). These data demonstrate that proliferation is active in the peripheral region of zebrafish lens epithelium.

### Cell-division orientation shifts from longitudinal to circumferential along the AP axis

**Fig. 4.**
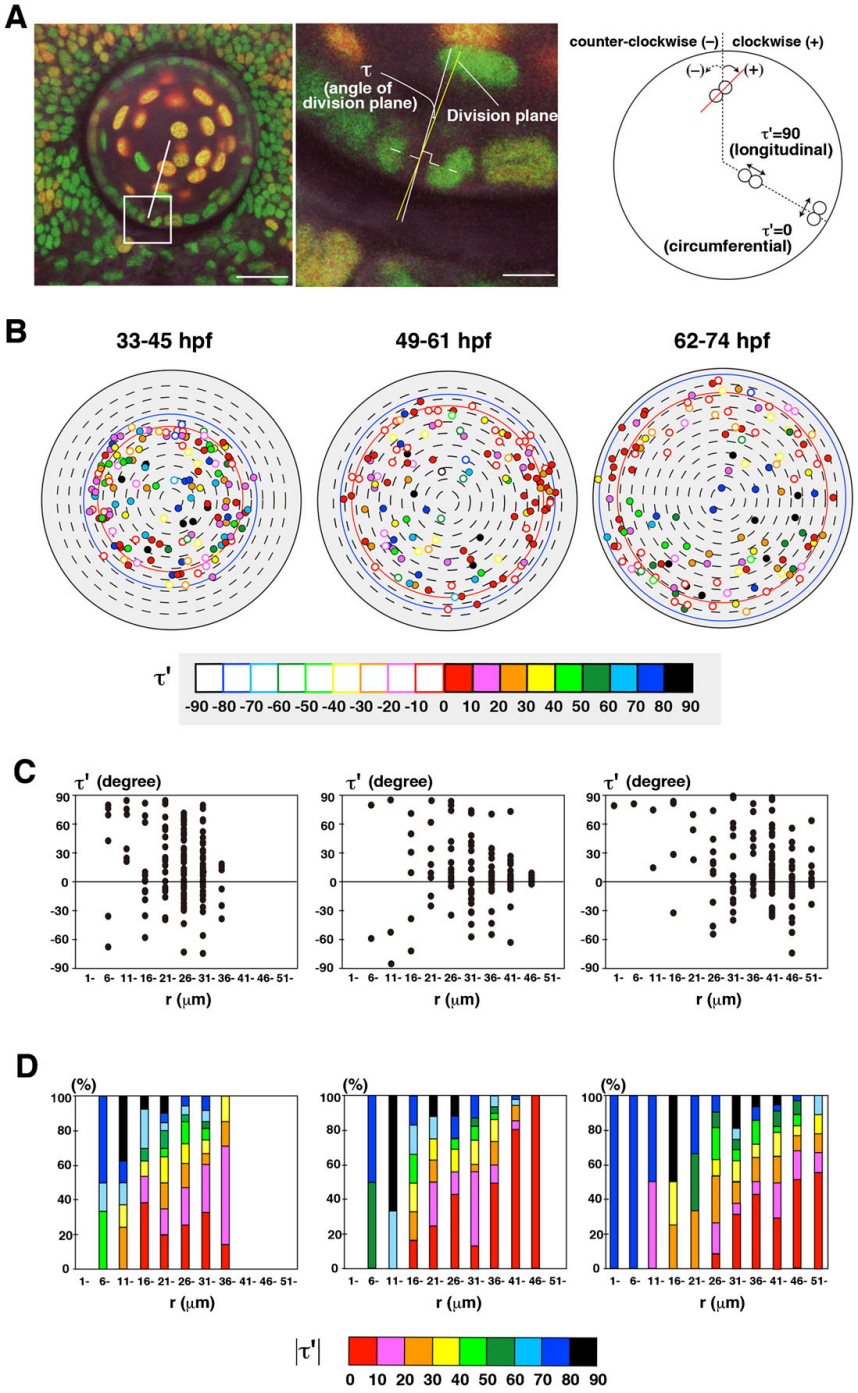
Cell-division orientation in zebrafish lens epithelium. (A) Measurement of the orientation of cell division. Cell-division orientation is defined as τ′, the angle between the line connecting segregating daughter cell chromatins and the circumferential line of the lens sphere. τ′ was calculated from τ, the angle between the r-axis and the cell-division plane, which was perpendicular to the line connecting segregating chromatins (see Materials and Methods). τ′ = 0 and 90 indicate circumferential and longitudinal cell division, respectively. Plus and minus indicate clockwise and counter-clockwise orientation, respectively. (B) Plotting of cell-division orientation in a projection view of the lens epithelium at three time windows: 33–45, 49–61, 62–74 hpf. Samples of mitoses were the same as shown in Fig. 3A. Cell-division orientation is indicated by color codes red (τ′ = 0–10), pink (τ′ = 11–20), orange (τ′ = 21–30), yellow (τ′ = 31–40), light green (τ′ = 41–50), dark green (τ′ = 51–60), light blue (τ′ = 61–70), blue (τ′ = 71–80), and black (τ′ = 81–90). Closed and open circles indicate clockwise and counter-clockwise orientations, respectively. (C) Plotting of τ′ of individual mitotic cells along the r-axis. Mitotic cells with low τ′ value (−30<τ′<30) is zero in the most anterior region (0<r<10), but increased in the peripheral region. Furthermore, the number of cells with the positive value of τ′ is higher than that of the negative value in all stages. (D) Histogram of absolute τ′ value along the r-axis. The fraction of cells with circumferential cell division is low in the anterior region, but gradually increased in a peripheral direction, and was dominant in the peripheral region. Scale bars: 20 µm (A, left panel), 5 µm (A, right panel).

Cell-division orientation is a key regulator to shape tissues (Gillies and Cabernard, 2011). We examined cell-division orientation in zebrafish lens epithelium. During mitosis, condensed chromosomes aligned along the cell-division plane and segregated in opposing directions within 30 min after alignment (Fig. 1D). We defined the cell-division orientation τ′ as the angle between the line that connects with two daughter nuclei after segregation and the circumferential line crossing the mother nucleus, which is perpendicular to the meridian (longitudinal) line of the lens sphere (see Materials and Methods). τ′ was calculated from τ, the angle between the line that connects two segregated daughter nuclei and the tangential line crossing the mother nucleus on the projection plane view (see Materials and Methods), which was measured as the angle between the division plane and the radial line (left two panels of Fig. 4A). In this definition, τ′ = 0 represents circumferential cell division, whereas τ′ = 90 represents longitudinal cell division (right panel of Fig. 4A). In this configuration, 0<τ′<90 and −90<τ′<0 indicates that cell-division orientation is along clockwise and counter-clockwise spiraling from the pole of the lens sphere, respectively. Thus, we refer to cell division with 0<τ′<90 and −90<τ′<0 as clockwise- and counter-clockwise-cell division, respectively.

Using the same time-lapse scanning data shown in Fig. 3, we determined the cell-division orientation of mitoses occurring in three periods: 33–45, 49–61, and 62–74 hpf. In all three periods, longitudinal cell-division orientation was prominent in the anterior region, whereas circumferential cell-division orientation was prominent in the peripheral region (Fig. 4B). The number of clockwise cell divisions was relatively higher than that of counter-clockwise, although both types were observed along the AP axis in all three stages (Fig. 4C; supplementary material Fig. S8A). We also found that the balance between clockwise and counter-clockwise orientation varied between lenses (supplementary material Fig. S8B). Plotting of the absolute value of τ′ clearly revealed that cell-division orientation shifted from longitudinal to circumferential along the AP axis of the lens epithelium (Fig. 4D). Lastly, to confirm that circumferential bias of cell-division orientation in the peripheral region is not due to an artifact of angle τ measurements and mathematical calculations, we directly measured angle τ′ in the peripheral region on the scanning image acquired from a ventral view of the lens from 33–45 hpf (supplementary material Fig. S9A). We confirmed that a fraction of lens epithelial cells with circumferential cell division (|τ′|<40) was around 80% at r = 31–35 µm and more than 60% at r = 36–40 µm, suggesting that circumferential cell division is dominant in the peripheral region (supplementary material Fig. S9B). These data suggest that cell-division orientation is spatially regulated in zebrafish lens epithelium.

### Long-axis orientation correlates with cell-division orientation in zebrafish lens epithelium

**Fig. 5.**
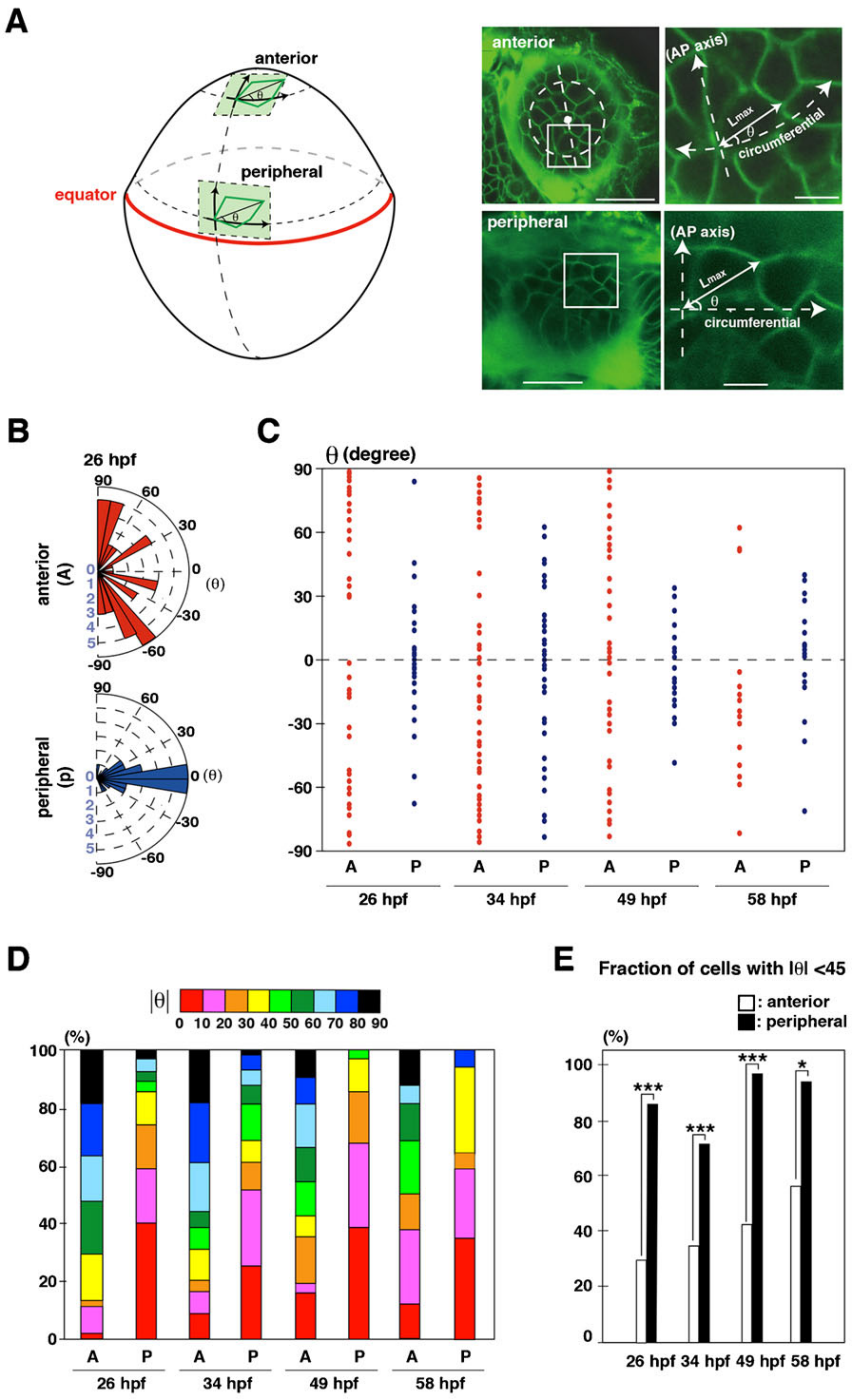
Orientation of the long axis of lens epithelial cells. (A) The orientation of the long axis is defined as the angle θ between the long axis and the circumferential line of the lens sphere. Plus and minus values indicate clock-wise and counter-clockwise directions. (B) Graph of the long-axis orientation at 26 hpf. Red and blue bars indicate the number of cells with a particular range of angle in the anterior and peripheral region, respectively. Longitudinal orientation is prominent in the anterior region, whereas circumferential orientation is prominent in the peripheral region. (C) Plotting of θ at 26, 34, 49 and 58 hpf. Red and blue dots represent individual epithelial cells in the anterior and peripheral regions, respectively. Circumferential orientation (−45°<θ<45°) is prominent in peripheral region. There seems to be no bias between the clockwise and counter-clockwise directions. (D) Histogram of absolute θ. Long-axis orientation is indicated by color codes. 30–50% of lens epithelial cells have θ less than 40° in the anterior region, but 70–95% do in the peripheral region, suggesting that longitudinal and circumferential orientations are prominent in anterior and peripheral regions, respectively. (E) Percentage of the number of lens epithelial cells whose long axis is circumferentially oriented (−45°<θ<45°). The long axis is circumferentially oriented in the peripheral region through all stages. Probability was calculated by χ^2^-test: *p<0.05, ***p<0.005. Scale bars: 20 µm (A, left panels), 5 µm (A, right panels).

It was proposed that cells tend to divide along their long axis, which is called “the long-axis rule” or “Hertwig rule” (Hertwig, 1884). On the other hand, cell division during gastrulation in zebrafish does not follow the Hertwig rule (Gong et al., 2004). We examined long-axis orientation in zebrafish lens epithelium. We visualized the apical geometries of individual cells by labeling with a fluorescent lipid dye, Bodipy ceramide. Because lens epithelium is thin, double labeling of lens epithelium with Bodipy ceramide and mCherry-fused zebrafish tight junction protein ZO1 (tjp1a) confirmed that Bodipy ceramide-positive lines conform to apical cell membranes labeled with mCherry-tjp1a (supplementary material Fig. S10). We defined the orientation of the long axis as θ, the angle between the long axis and the circumferential line (Fig. 5A) and measured θ in the anterior and peripheral regions. For this analysis, we divided the anterior region into four different areas, nasal, temporal, dorsal, and ventral, relative to the anterior pole of the lens sphere (supplementary material Fig. S11A). First, we examined long-axis orientation in the antero-ventral region as a representative of the anterior region. Long-axis orientation was longitudinally biased in the anterior region and circumferentially biased in the peripheral region (Fig. 5B). There seemed to be no clockwise bias in either region (Fig. 5C). Plotting the absolute value of θ clearly revealed that the long-axis orientation was longitudinally and circumferentially biased in the anterior and peripheral regions, respectively, from 26 to 58 hpf (Fig. 5D), suggesting that the long-axis orientation is spatially regulated in zebrafish lens epithelium. The fraction of cells with θ<45 in the peripheral region was significantly higher than that of the anterior region from 26 to 58 hpf (Fig. 5E).

Next, we confirmed that the longitudinal bias of long-axis orientation was observed in all four anterior subdomains (nasal, temporal, dorsal and ventral areas) (supplementary material Fig. S11B). The fraction of cells with θ<45 was less than 50% in all four areas and they were significantly lower than in the peripheral region (supplementary material Fig. S11C). Taken together, these data suggest that long-axis orientation correlates with cell-division orientation in zebrafish lens epithelium.

### Other apical cell geometry characters in lens epithelium

We examined other apical cell geometry characters: cell shape uniformity, vertex number, and size. We examined cell shape uniformity by measuring the ratio of the long axis relative to the short axis. The ratio was higher in the peripheral region than in the anterior region at all stages, and the difference was significant at 34, 49, and 77 hpf, suggesting that lens epithelial cells elongate circumferentially in the peripheral region (supplementary material Fig. S12). Next, we counted the number of vertices of individual lens epithelial cells (supplementary material Fig. S13A). The fraction of lens epithelial cells with 6 vertices and above in the peripheral region was more than 60% after 49 hpf, and higher than those in the anterior region at all stages (supplementary material Fig. S13B). Average vertex number was also significantly higher in the peripheral region than in the anterior-most region after 49 hpf (supplementary material Fig. S13C). Next, we examined apical size by measuring the total perimeter length of individual cells. The total apical perimeter length was higher in the peripheral region than in the anterior region after 49 hpf (supplementary material Fig. S14), suggesting that apical size in the peripheral region exceeds that of the anterior region after 49 hpf. Thus, lens epithelial cells elongate circumferentially to increase the fraction of hexagonal cells and their perimeter length in the peripheral region, which is reminiscent to cross section pattern of lens fiber cells.

### E-cadherin is expressed in lens epithelium

Our findings demonstrated that apical cell geometry and cell division are spatially regulated in zebrafish lens epithelium. It has been reported that cell adhesion influences cell geometry and cell-division orientation in human HeLa cells (Théry et al., 2007; Théry et al., 2005; Toyoshima and Nishida, 2007). Here we focused on a cell adhesion molecule, E-cadherin. In vertebrates, E-cadherin is expressed in lens epithelium (Pontoriero et al., 2009; Xu et al., 2002). To clarify the spatial relationship between E-cadherin expression and the peripheral proliferating zone, we compared *e-cadherin* mRNA expression and BrdU incorporation. BrdU was mainly incorporated in the peripheral proliferating zone. *e-cadherin* mRNA was expressed in the lens epithelium, but low in the peripheral proliferating zone (supplementary material Fig. S15A–C). Pax6 is a marker of lens epithelium in zebrafish (Imai et al., 2010). Double labeling with anti-Pax6 and anti-E-cadherin antibodies revealed that Pax6 was expressed, but E-cadherin expression was reduced in the peripheral proliferating zone (supplementary material Fig. S15D–F). These data suggest that E-cadherin is expressed in the anterior region of the lens epithelium and reduced in the peripheral proliferating zone.

### Lens fiber cell differentiation proceeds in a hypomorphic allele of an E-cadherin mutant

To examine the role of E-cadherin in zebrafish lens epithelial cells, we examined lens phenotypes in a zebrafish *e-cadherin* mutant, *hab*. Kane et al. reported that gastrulation is compromised in *hab* mutants (Kane et al., 1996). However, when a hypomorphic allele of *e-cadherin* mutant, *hab*^rk3^, is maintained at 31°C, *hab*^rk3^ mutant embryos undergo normal gastrulation (Shimizu et al., 2005). In 31°C-bred *hab*^rk3^ mutant embryos, lens epithelium normally expressed a lens epithelial marker, Pax6, and was maintained as monolayer at least until 48 hpf, although E-cadherin protein expression was absent at 36 hpf and weaker at 48 hpf (supplementary material Fig. S16). Furthermore, markers of lens fiber cell differentiation, Aquaporin 0 (AQP0) and Prox1, were normally expressed in 31°C-bred *hab*^rk3^ mutant embryos at 48 and 72 hpf (supplementary material Fig. S17), suggesting that lens fiber cell differentiation normally proceeds in this allele at least until 72 hpf.

Next, we examined whether cell proliferation is normal in *hab*^rk3^ mutant lens epithelium. BrdU incorporation in wild type and *hab*^rk3^ mutants at 48 and 72 hpf revealed that cell proliferation was maintained in *hab*^rk3^ mutant lens epithelium (supplementary material Fig. S18A–D). We examined the fraction of BrdU-positive cells in Pax6-positive lens epithelial cells, and found that the BrdU-positive cell fraction was slightly lower in *hab*^rk3^ mutants than in wild-type siblings, but not statistically significant (supplementary material Fig. S18E). These data suggest that lens epithelial cell proliferation and lens fiber cell differentiation normally proceeded in *hab*^rk3^ mutant at least until 72 hpf.

### E-cadherin regulates cell-division orientation in lens epithelium

**Fig. 6.**
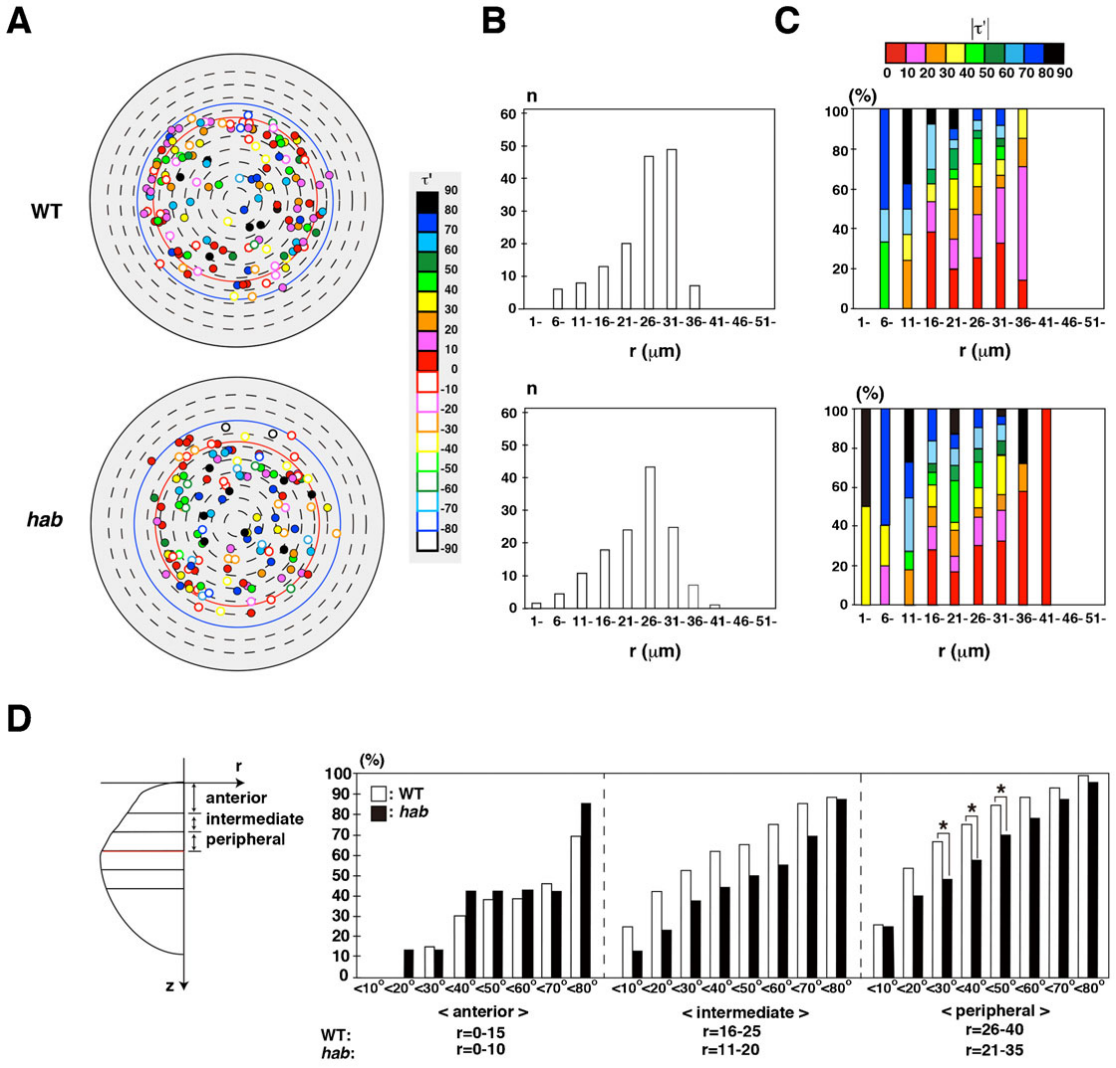
E-cadherin is required for circumferential orientation of cell division. (A) Mitotic positions in lens epithelium of wild type and *hab*^rk3^ mutants for 33–45 hpf. Wild-type data were the same as in Fig. 4B. All mitoses of *hab*^rk3^ mutants were merged from three lenses, shown in supplementary material Fig. S2. Cell-division orientation is indicated by color codes. Filled and open circles indicate clockwise and counter-clockwise orientations, respectively. Red and blue lines indicate the equator of the smallest lenses (r = 31 µm) at the beginning and the largest lens (r = 40 µm) at the end of the scanned period, respectively. (B) Number of mitoses along the r-axis. (C) Histogram of cell-division orientation (absolute value of τ′) along the r-axis. Cell-division orientation is indicated by color codes. (D) Comparison of percentage of cell-division orientation with values of absolute τ′ between wild type and *hab*^rk3^ mutants. Lens epithelium is divided into anterior, intermediate, and peripheral regions. Cell divisions with |τ′|<30, |τ′|<40, and |τ′|<50 are lower in the peripheral region of *hab*^rk3^ mutants than in wild type. Probability was calculated by χ^2^-test: *p<0.05.

Next, we combined *hab*^rk3^ mutants with the transgene, *Tg(h2afv:GFP; EF1α:mCherry-zGem)*, and examined cell-division orientation in lens epithelium during the stage from 33 to 45 hpf (Fig. 6A; supplementary material Movie 7 for 3D image at 33 hpf). Here we used three *hab*^rk3^ mutant lenses (supplementary material Fig. S2A) and confirmed that the equatorial position was within 31–36 µm at the beginning and not drastically changed during the scanned period (supplementary material Fig. S2B). As in wild type, the number of mitotic cells was low in the anterior region and high in the peripheral region in *hab*^rk3^ mutants (Fig. 6B), suggesting that proliferation is active in the peripheral region in *hab*^rk3^ mutants. Like wild type, cell-division orientation was shifted from longitudinal to circumferential directions along the AP axis in *hab*^rk3^ mutants (Fig. 6C). To compare cell division orientation more precisely between wild type and *hab*^rk3^ mutants, we divided lens epithelium into three regions: anterior, intermediate and peripheral, by normalizing lens size differences between wild type and *hab*^rk3^ mutants (see Materials and Methods). The fraction of mitotic cells with circumferential division orientation was decreased in the intermediate and peripheral region of *hab*^rk3^ mutant; in particular, the fraction with absolute τ′ less than 30°, 40°, and 50° was significantly lower in the peripheral region of *hab*^rk3^ mutant relative to the wild type (Fig. 6D). These data suggest that cell-division orientation is more longitudinally biased in *hab*^rk3^ mutant lens epithelium, especially in the peripheral region. Thus, E-cadherin is required for the circumferential bias of cell-division orientation in the peripheral region of lens epithelium.

### E-cadherin regulates cell geometry in lens epithelium

**Fig. 7.**
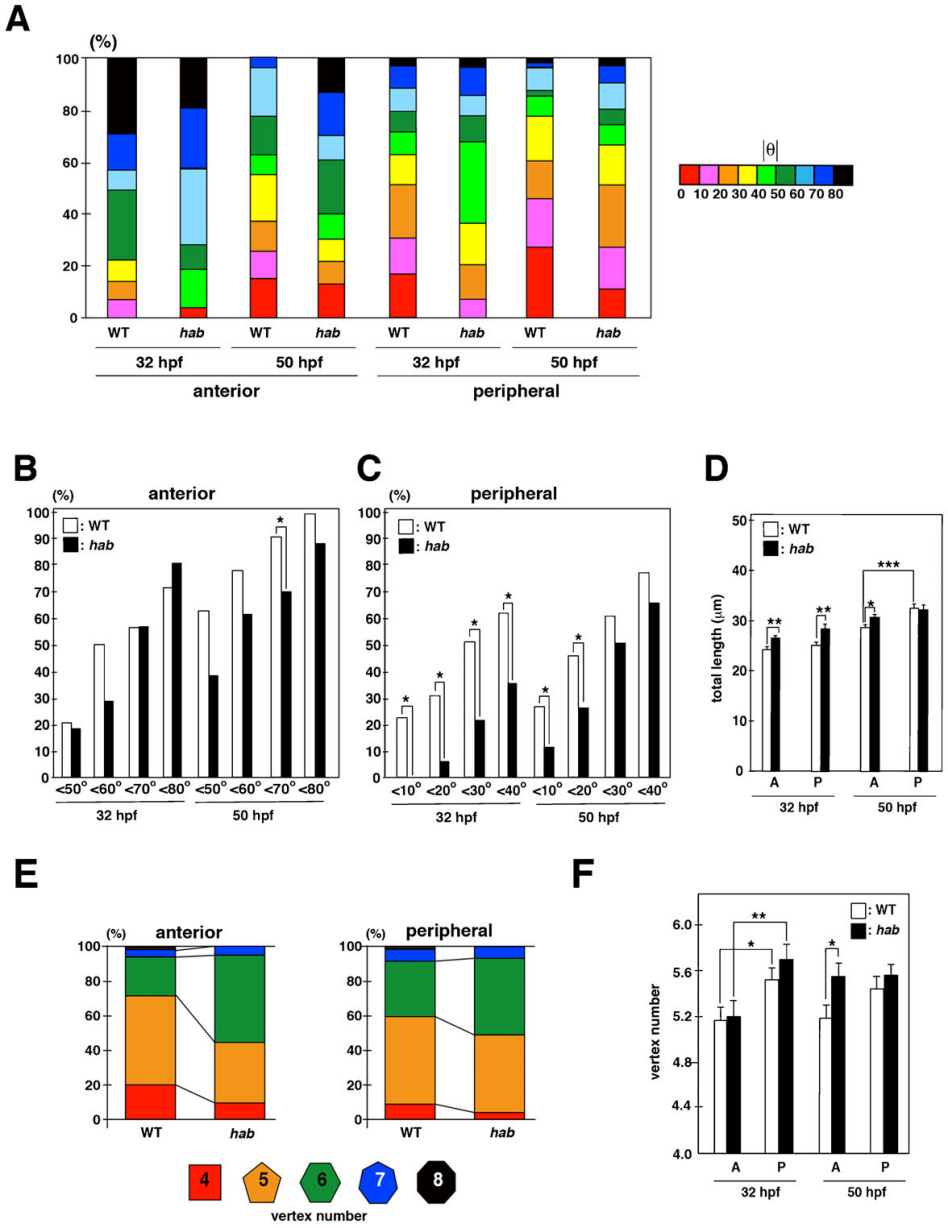
E-cadherin is required for circumferential orientation of the long axis. (A) Histogram of long-axis orientation in wild-type sibling and *hab*^rk3^ mutants at 32 and 50 hpf. (B,C) Comparison of the percentage of long-axis orientation with different values of absolute θ between wild-type sibling and *hab*^rk3^ mutants at 32 and 50 hpf in anterior (B) and peripheral (C) regions. Fraction of the long axis θ<70 is significantly higher in wild-type sibling than in *hab*^rk3^ mutants at 50 hpf (B). The fraction of the circumferentially biased long axis (θ<40 at 32 hpf and θ<20 at 50 hpf) is higher in wild-type sibling than in *hab*^rk3^ mutants (C). Probability was calculated by χ^2^-test: *p<0.05. (D) Average perimeter length of lens epithelial cells in the anterior and peripheral regions. In wild type, perimeter length is longer in the peripheral region than in the anterior region at 50 hpf. Perimeter length is longer in *hab*^rk3^ mutants than in wild type in both anterior and peripheral regions at 32 hpf and in the anterior region at 50 hpf. Bars and lines indicate average and standard error. t-test: *p<0.05, **p<0.01, ***p<0.005. (E) Percentage of vertex number of lens epithelial cells in the anterior and peripheral regions at 50 hpf. (F) Average vertex number in the anterior and peripheral regions. Vertex number is higher in the peripheral region than in the anterior region of wild-type sibling and the *hab*^rk3^ mutants at 32 hpf. Vertex number in both anterior and peripheral regions is not statistically different between wild type and the *hab*^rk3^ mutants at 32 hpf. At 50 hpf, vertex number in the anterior region is higher in *hab*^rk3^ mutants than in wild-type sibling, whereas vertex number in the peripheral region is not different between wild-type sibling and *hab*^rk3^ mutants. Bars and lines indicate average and standard error. t-test: *p<0.05, **p<0.01.

We examined the orientation of the long axis in *hab*^rk3^ mutant lens epithelium. Since there was no bias between clockwise and counter-clockwise orientation in long-axis orientation in wild-type lens, we examined the absolute value of θ (Fig. 7A). In the anterior region, the fraction of cells with θ<70 was significantly lower in *hab*^rk3^ mutants than in wild type at 50 hpf (Fig. 7B), suggesting that long-axis orientation is more longitudinally shifted in *hab*^rk3^ mutants. In the peripheral region, the fraction of cells with θ<40 was significantly lower in *hab*^rk3^ mutant than in wild type at 32 hpf, and the fraction of cells with θ<20 was significantly lower in *hab*^rk3^ mutant than in wild type at 50 hpf (Fig. 7C), suggesting that long-axis orientation is more longitudinally shifted in *hab*^rk3^ mutants in the peripheral region. These data suggest that orientation of the long axis and cell division are more longitudinally biased in *hab*^rk3^ mutants.

We examined cell size by measuring total perimeter length. As is consistent with the data on wild type perimeter length (supplementary material Fig. S14), in wild-type sibling, total length in the peripheral region was not different from that of the anterior regions at 32 hpf, but significantly higher than that of the anterior regions at 50 hpf (Fig. 7D). At 32 hpf, total length was slightly but significantly larger in both anterior and peripheral regions of *hab*^rk3^ mutants than in wild-type sibling (Fig. 7D). At 50 hpf, total length was larger only in the anterior region of *hab*^rk3^ mutants than in wild-type sibling (Fig. 7D). There was no difference in total perimeter length between the anterior and peripheral regions in *hab*^rk3^ mutants at 50 hpf. These data suggest that cell apical size increases especially in the anterior region of lens epithelium in the absence of E-cadherin.

We examined vertex number in *hab*^rk3^ mutants. In the anterior region at 50 hpf, lens epithelial cells with 5 vertexes and below constituted nearly 70% of all cells in wild type, but only 45% in *hab*^rk3^ mutants (Fig. 7E). In the peripheral region at 50 hpf, lens epithelial cells with 5 vertexes and below comprised 60% of all cells in wild type, but only 50% in *hab*^rk3^ mutants (Fig. 7E). The average vertex number was not significantly different between *hab*^rk3^ mutants and wild-type sibling in the anterior and peripheral regions at 32 hpf (Fig. 7F). In both wild-type sibling and *hab*^rk3^ mutants, the average vertex number in the peripheral region is higher than that of the anterior region at 32 hpf. At 50 hpf, the average vertex number in anterior region was significantly higher in *hab*^rk3^ mutants than in wild-type sibling (Fig. 7F). The average vertex number was not significantly different between *hab*^rk3^ mutants and wild-type sibling in the peripheral region. Since the fraction of hexagonal lens epithelial cells was increased in *hab* mutant at 50 hpf (Fig. 7E), we examined whether the side length of hexagonal cells is decreased in *hab* mutant. The average side length of hexagonal cells was shorter in the *hab* mutant than in wild type, in the peripheral region at 32 hpf and in both the anterior and peripheral regions at 50 hpf (supplementary material Fig. S19). In particular, the difference was statistically significant in the peripheral region at 50 hpf (supplementary material Fig. S19). These data suggest that reduced cell adhesive activity decreases the length of each side, resulting in increased apical cell size with higher vertexes.

## DISCUSSION

The Fucci system is a powerful method to visualize cell-cycle phases in cultured human cells and in mouse and zebrafish embryos (Sakaue-Sawano et al., 2008; Sugiyama et al., 2009). However, cell-cycle progression is fast and G1 phase is short in zebrafish embryos. It is less efficient to detect mKO2-zCdt1 in highly proliferating tissues such as the retina after 30 hpf in zebrafish. Furthermore, transcription by the *EF1α* promoter declines in later stages in zebrafish, resulting in reduced capacity to detect mAG-zGem in S phase. In this study, we established the zebrafish transgenic line *Tg(h2afv:GFP; EF1α:mCherry-zGem)* to overcome these problems. This transgenic line expresses mCherry-zGem, the fluorescence of which is stronger than that of mAG-zGem, and GFP-Histone 2A, which visualizes chromatin through all cell-cycle phases. This transgenic line enables us to distinguish cell-cycle phases even in rapidly proliferating tissue until 72 hpf. Furthermore, we can measure cell-division orientation in time-lapse scanning images. Thus, *Tg(h2afv:GFP; EF1α:mCherry-zGem)* is useful to observe cell-cycle progression and cell division *in vivo*.

In this study, we examined spatial and temporal profiles of cell-cycle progression of zebrafish lens epithelium, and found that cell proliferation is active in the peripheral zone anterior to the equator. Such a proliferating zone, called the germinative zone, was reported in chick and mammalian lenses (McAvoy, 1978; Modak et al., 1968; Zhou et al., 2006). Our findings suggest that the germinative zone is conserved in zebrafish. Furthermore, cell-division orientation is longitudinally biased in the anterior region, shifted from the longitudinal to the circumferential direction along the AP axis, and circumferentially biased in the peripheral region. These data suggest that cell-division orientation is spatially regulated in zebrafish lens epithelium.

What is the biological significance of the spatial pattern of cell-division orientation in the lens epithelium? In the developing lens, the equator functions as a platform where lens epithelial cells start to differentiate into lens fiber cells (Mochizuki and Masai, 2014). The frequency with which lens epithelial cells pass through the equator must be uniform along the equatorial circumference to maintain a spheroidal shape during lens development. Cell division increases the number of lens epithelial cells, which subsequently increases tension between lens epithelial cells. It is likely that such high tension causes lens epithelial cells to migrate toward the equator. We often observed that, after cell division in the peripheral zone, one of two daughter cells migrated toward the equator but the other stayed in the circumferential proliferating zone (unpublished data). One possibility is that circumferential-biased cell-division in the germinative zone keeps daughter cells in germinative zone and increases tension between lens epithelial cells along the equatorial circumference, which promotes uniform migration of lens epithelial cells through the equator. An alternative possibility is that lens epithelial cells passing through the equator reduce tension heterogeneously in the germinative zone, which may be negated by circumferential cell division. In both cases, circumferential cell division may buffer adequate number of lens epithelial cells and keep appropriate tension in the germinative zone. If this is the case, coupling of circumferential cell division with tension-mediated cell migration may enable lens epithelial cells to enter lens fiber differentiation with appropriate frequency.

In this study, we show that cell-division orientation correlates with long-axis orientation in zebrafish lens epithelium, suggesting that lens epithelial cells follow the Hertwig rule. Furthermore, E-cadherin is required for circumferential shift of the long axis and cell-division orientation in zebrafish lens epithelium. In E-cadherin mutants, apical cell size is larger and vertex number is increased in anterior lens epithelium. These data suggest that reduced cell adhesion compromises packing of lens epithelial cells. It is likely that cell adhesion-mediated epithelial packing requires a spatial pattern of apical cell geometry and cell-division orientation in the germinative zone. E-cadherin may buffer tensile fluctuation between lens epithelial cells through its cell adhesive activity and biasing cell-division orientation.

In summary, we show a spatial correlation of E-cadherin, apical cell shape, and cell-division orientation in zebrafish lens epithelium. In the developing lens, cell division seems to be a major driving force that promotes epithelial cells to migrate in an anterior to peripheral direction. Preliminary observations suggest that lens epithelial cells migrate not along the longitudinal line of the lens sphere, but rather in oblique directions in the peripheral region of zebrafish lens epithelium (unpublished data). In the future, it is important to elucidate whether cell-division orientation plays a role in the migration of lens epithelial cells and spheroidal growth of lens spheres.

## Supplementary Material

Supplementary Material
