# Supplementary material for: Spatial pattern of cell geometry and cell-division orientation in zebrafish lens epithelium
Source: Biol Open. 2014 Sep 26;3(10):982–94. doi: 10.1242/bio.20149563 (PMC4197447; doi:10.1242/bio.20149563)
Supplement: Supplementary Material [file supp_3_10_982__index.html]

Spatial pattern of cell geometry and cell-division orientation in zebrafish lens epithelium — Spatial pattern of cell geometry and cell-division orientation in zebrafish lens epithelium — Supplementary Material 

# Spatial pattern of cell geometry and cell-division orientation in zebrafish lens epithelium

## bio.20149563 Supplementary Material

**Files in this Data Supplement:**

- Supplementary Material - Toshiaki Mochizuki et al. doi: 10.1242/bio.20149563
- Movie 1 - **Movie 1. 3D scanning of 37 hpf wild type lenses combined with zebrafish transgenic line *Tg(h2afv:GFP; EF1α:mCherry-zGem)*.**
- Movie 2 - **Movie 2. 3D scanning of 49 hpf wild type lenses combined with zebrafish transgenic line *Tg(h2afv:GFP; EF1α:mCherry-zGem)*.**
- Movie 3 - **Movie 3. 3D scanning of 62 hpf wild type lenses combined with zebrafish transgenic line *Tg(h2afv:GFP; EF1α:mCherry-zGem)*.**
- Movie 4 - **Movie 4. Cell-cycle progression of lens epithelial cells from S to M phases in zebrafish transgenic line *Tg(h2afv:GFP; EF1α:mCherry-zGem)*.**
- Movie 5 - **Movie 5. Mitoses of wild type lens epithelial cells in zebrafish transgenic line *Tg(h2afv:GFP; EF1α:mCherry-zGem)*.**
- Movie 6 - **Movie 6. Time-lapse movie of anterior region of wild-type lens combined with zebrafish transgenic line *Tg(h2afv:GFP; EF1α:mCherry-zGem)* from 33 to 45 hpf.**
- Movie 7 - **Movie 7. 3D scanning of *hab*rk3 mutant lenses combined with zebrafish transgenic line *Tg(h2afv:GFP; EF1α:mCherry-zGem)* at 33 hpf.**
