# Supplementary material for: Spatial pattern of cell geometry and cell-division orientation in zebrafish lens epithelium
Source: Biol Open. 2014 Sep 26;3(10):982–94. doi: 10.1242/bio.20149563 (PMC4197447; doi:10.1242/bio.20149563)
Supplement: Supplementary Material [file supp_bio.20149563_bio.20149563-s1.pdf]

Supplementary Material

Toshiaki Mochizuki et al. doi: 10.1242/bio.20149563

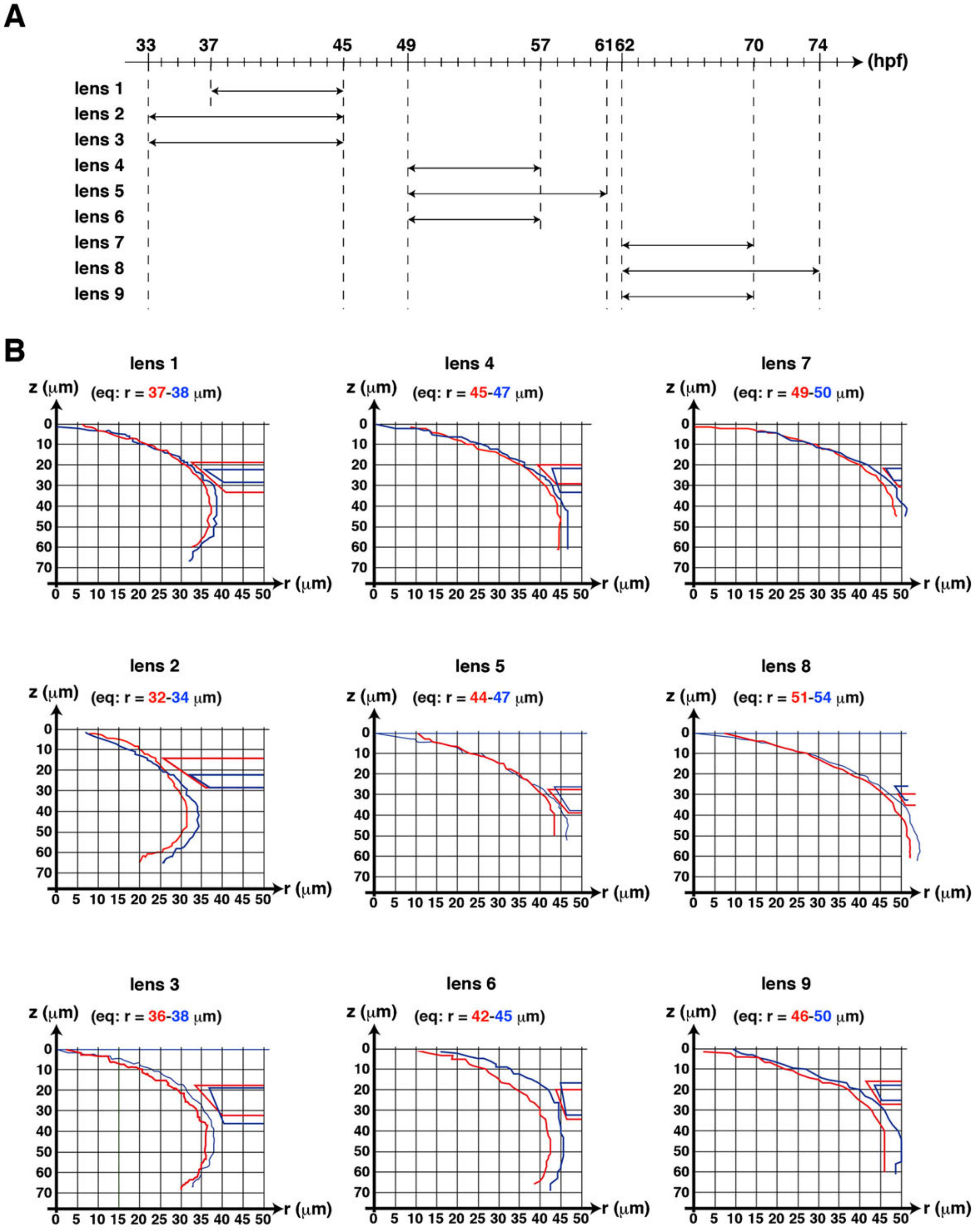

Fig. S1. Wild-type lenses used in the experiments shown in Figs 3, 4, and 6. (A) Scanning of nine wild-type lenses. Lenses 1–3 were used for scanning during the time window 33–45 hpf, lenses 4–6 for 49–61 hpf, and lenses 7–9 for 62–74 hpf. (B) Sizes of individual lenses at the beginning (red) and the end (blue) of the scanned period are shown in the r–z coordinate. Positions of the retinal ciliary marginal zone that was associated with lens epithelium at the beginning and the end of the scanned period are shown as red- and blue-colored trapezia, respectively. In most cases, lenses grew. The equatorial position of an individual lens is shown.

Biology Open (2014) 000, 1–13 doi:10.1242/bio.20149563

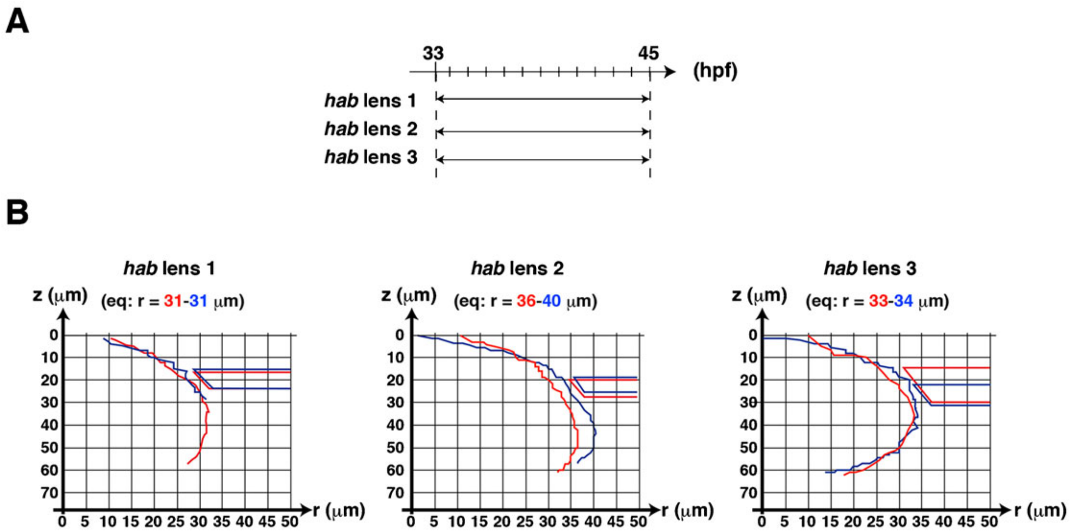

Fig. S2. ***hab*rk3 mutant lenses used in the experiments shown in Fig. 6.** (A) Scanning period of three *hab*rk3 mutant lenses. Lenses 1–3 were used for scanning during the time window 33–45 hpf. (B) Sizes of individual lenses at the beginning (red) and the end (blue) of the scanned period are shown in the r–z coordinate. Positions of the retinal ciliary marginal zone that was associated with lens epithelium at the beginning and the end of the scanned period are shown as red and blue trapezia, respectively.

Biology Open

Biology Open (2014) 000, 1–13 doi:10.1242/bio.20149563

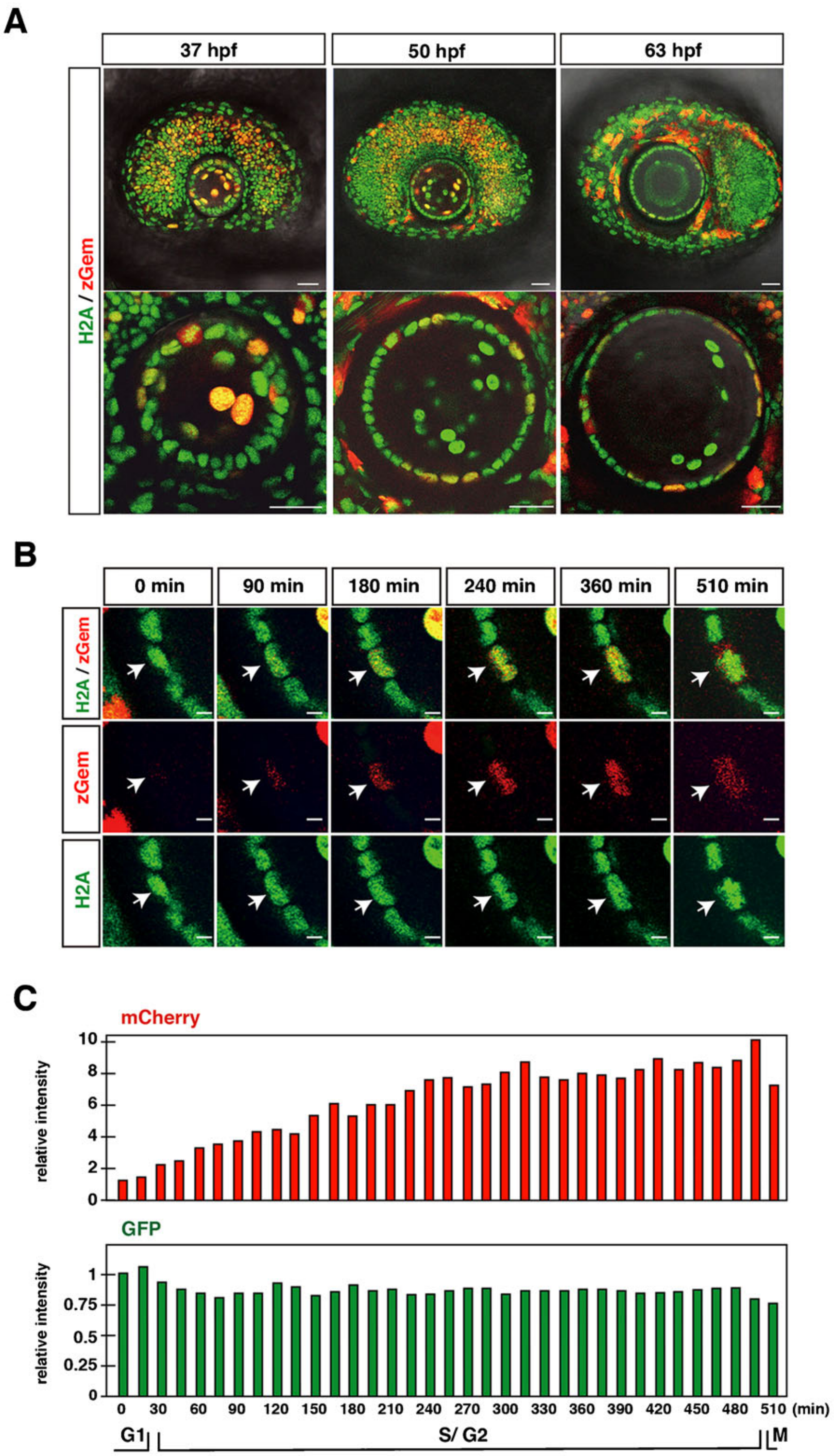

Fig. S3. Transgenic zebrafish *Tg(h2afv:GFP; EF1α:mCherry-zGem)*. (A) Confocal scanning of the neural retina and lens in a zebrafish transgenic line *Tg(h2afv:GFP; EF1α:mCherry-zGem)* at 37, 50 and 63 hpf. Bottom panels indicate higher magnification of the lens. (B) Time-lapse observations of mCherry and GFP expression in lens epithelial cells undergoing S and G2 phase (arrow) in the *Tg(h2afv:GFP; EF1α:mCherry-zGem)* lens. mCherry expression gradually increases during S phase and reaches a plateau at 360 min. Then it begins to be down-regulated during M phase (510 min). (C) Intensity of mCherry and GFP florescence in the *Tg(h2afv:GFP; EF1α:mCherry-zGem)* lens during S and G2 phases. Intensity of mCherry and GFP is normalized by initial fluorescent intensity at t=0. Scale bars: 20 μm (A), 2 μm (B).

Biology Open

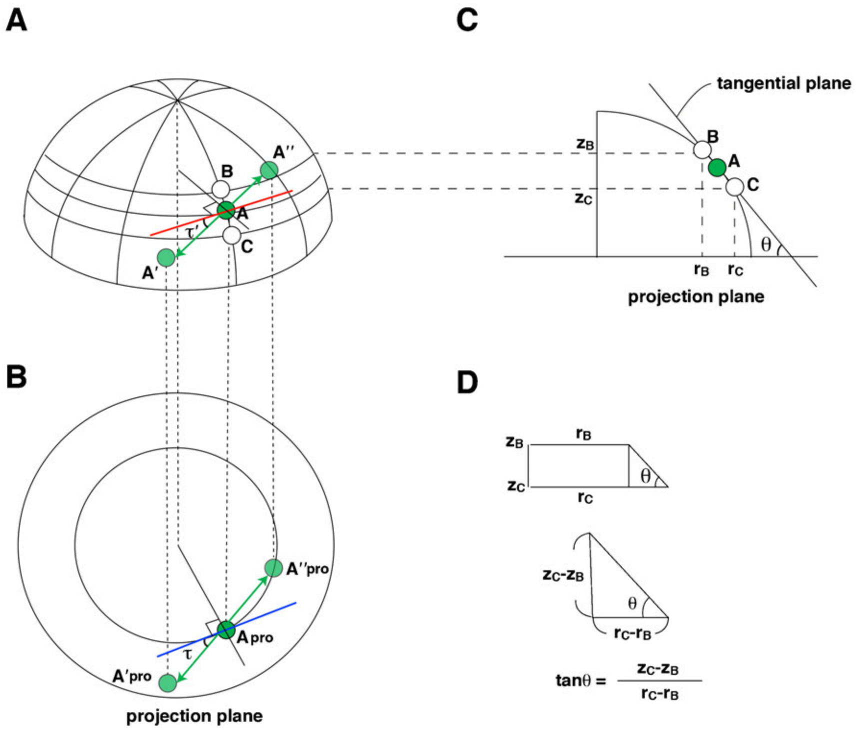

Fig. S4. Calculation of the cell-division orientation. (A) Schematic drawing of the anterior region of the lens. Green circles indicate a mother cell, A, and two daughter cells A′ and A″. B and C are cells located at the anterior and posterior to cell A along the longitudinal line. Cell-division orientation is indicated by τ', the angle between the circumference (red line) that crosses the mother cells A and the line that connects nuclei of A′ and A″. (B) The projection plane containing mother cell A. A'_{pro} and A''_{pro} indicate projected positions of daughter cells A′ and A″. τ, indicates the angle between the line that connects A'_{pro} and A''_{pro} and the tangential line that crosses mother cell A (Blue line), and was determined by measurement of the angle between the cell-division plane and the radial axis (Fig. 4A). (C) θ is defined as the angle between the line that connects cells B and C and the horizontal plane. Their radius and z-position are defined as r_b, z_b, r_c, z_c, respectively. (D) θ was calculated by the equation: $\tan\theta = (z_c - z_b)/(r_c - r_b)$.

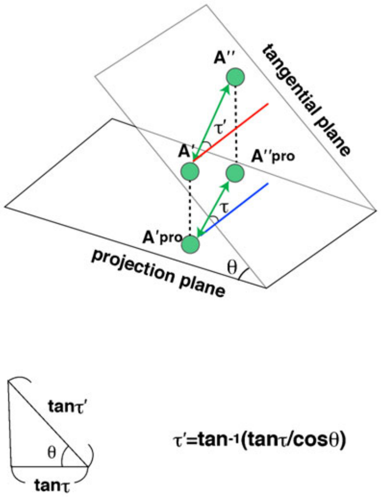

Fig. S5. Schematic drawing of τ and τ' on projection plane and tangential plane. τ' was calculated using the equation: $\cos\theta=\tan\tau'/\tan\tau$.

Biology Open (2014) 000, 1–13 doi:10.1242/bio.20149563

Fig. 2B–D

genotype	stage	lens number
wt	33–45 hpf	1

Fig. 3A, 4B, 6A

genotype	stage	lens number	cell number
wt	33–45 hpf	3	150
	49–61 hpf	3	129
	62–74 hpf	3	130

Fig. 6A

genotype	stage	lens number	cell number
hab	33–45 hpf	3	136

Fig. 5E

stage	anterior			peripheral			Chi-test	
	lens	cell	<45°	lens	cell	<45°	Chi-square value	p value
26 hpf	7	44	13	4	28	24	18.85	p<0.005
34 hpf	8	54	19	6	42	30	12.42	p<0.005
49 hpf	7	42	18	4	28	27	21.00	p<0.005
58 hpf	3	16	9	4	25	8	6.44	p<0.025

Fig. 6D

anterior

angle range	WT (lens=3)	hab (lens=3)	Chi-square test (wt vs hab)	
	cell number	cell number	Chi-square value	p-value
<10°	0	0		
<20°	0	1	2.10	p>0.05
<30°	2	1	0.00	p>0.05
<40°	4	3	0.43	p>0.05
<50°	5	3	0.10	p>0.05
<60°	5	3	0.10	p>0.05
<70°	7	3	0.10	p>0.05
<80°	10	6	0.53	p>0.05
<90°	14	7		

intermediate

angle range	WT (lens=3)	hab (lens=3)	Chi-square test (wt vs hab)	
	cell number	cell number	Chi-square value	p-value
<10°	8	4	1.07	p>0.05
<20°	14	7	2.30	p>0.05
<30°	17	11	1.15	p>0.05
<40°	21	13	2.20	p>0.05
<50°	22	15	1.43	p>0.05
<60°	25	16	2.92	p>0.05
<70°	28	20	2.23	p>0.05
<80°	29	25	0.04	p>0.05
<90°	33	29		

peripheral

angle range	WT (lens=3)	hab (lens=3)	Chi-square test (wt vs hab)	
	cell number	cell number	Chi-square value	p-value
<10°	27	23	0.04	p>0.05
<20°	55	37	3.39	p>0.05
<30°	68	44	6.58	p<0.025
<40°	77	53	6.43	p<0.025
<50°	87	64	4.84	p<0.05
<60°	91	72	3.61	p>0.05
<70°	96	80	2.16	p>0.05
<80°	102	88	2.22	p>0.05
<90°	103	92		

Fig. 7B, 7C

anterior 32 hpf

angle range	WT (lens=3)	hab (lens=5)	Chi-square test (wt vs hab)	
	cell number	cell number	Chi-square value	p-value
<10°	0	1	0.69	p>0.05
<20°	1	1	0.09	p>0.05
<30°	2	1	0.97	p>0.05
<40°	3	1	2.31	p>0.05
<50°	3	4	0.03	p>0.05
<60°	7	6	1.65	p>0.05
<70°	8	12	0.00	p>0.05
<80°	10	17	0.43	p>0.05
<90°	14	21		

peripheral 32 hpf

angle range	WT (lens=5)	hab (lens=4)	Chi-square test (wt vs hab)	
	cell number	cell number	Chi-square value	p-value
<10°	6	0	5.31	p<0.05
<20°	11	2	5.60	p<0.05
<30°	18	6	5.48	p<0.05
<40°	22	10	4.59	p<0.05
<50°	25	19	0.09	p>0.05
<60°	28	22	0.02	p>0.05
<70°	31	24	0.11	p>0.05
<80°	34	27	0.03	p>0.05
<90°	35	28		

anterior 50 hpf

angle range	WT (lens=7)	hab (lens=6)	Chi-square test (wt vs hab)	
	cell number	cell number	Chi-square value	p-value
<10°	4	3	0.03	p>0.05
<20°	7	3	1.29	p>0.05
<30°	10	5	1.38	p>0.05
<40°	15	7	3.18	p>0.05
<50°	17	9	2.83	p>0.05
<60°	21	14	1.69	p>0.05
<70°	26	16	6.60	p<0.025
<80°	27	20	3.75	p>0.05
<90°	27	23		

peripheral 50 hpf

angle range	WT (lens=8)	hab (lens=8)	Chi-square test (wt vs hab)	
	cell number	cell number	Chi-square value	p-value
<10°	15	6	4.19	p<0.05
<20°	26	14	4.69	p<0.05
<30°	34	27	1.05	p>0.05
<40°	43	35	2.10	p>0.05
<50°	48	39	2.49	p>0.05
<60°	49	43	0.83	p>0.05
<70°	54	48	1.56	p>0.05
<80°	55	51	0.40	p>0.05
<90°	56	53		

Fig. 7D

t-test

sample	lens	cell	WT 32 hpf A	WT 32 hpf P	hab 32 hpf A	hab 32 hpf P
WT 32 hpf A	8	56		0.240	0.010	
WT 32 hpf P	8	56				0.003
hab 32 hpf A	8	56				0.070
hab 32 hpf P	8	56				

t-test

sample	lens	cell	WT 50 hpf A	WT 50 hpf P	hab 50 hpf A	hab 50 hpf P
WT 50 hpf A	8	56		7.0x10-5	0.040	
WT 50 hpf P	8	56				0.770
hab 50 hpf A	8	56				0.230
hab 50 hpf P	8	53				

Fig. 7F

t-test

sample	lens	cell	WT 32 hpf A	WT 32 hpf P	hab 32 hpf A	hab 32 hpf P
WT 32 hpf A	8	68		0.037	0.781	
WT 32 hpf P	8	60				0.295
hab 32 hpf A	8	67				0.007
hab 32 hpf P	8	58				

t-test

sample	lens	cell	WT 50 hpf A	WT 50 hpf P	hab 50 hpf A	hab 50 hpf P
WT 50 hpf A	8	59		0.105	0.0151	
WT 50 hpf P	8	56				0.419
hab 50 hpf A	8	57				0.981
hab 50 hpf P	8	53				

Fig. S9

genotype	stage	lens number	cell number
wt	33–45 hpf	3	26

Fig. S11

region	lens	cell	<45°	Chi-square value				
				AV	AN	AD	AT	P
anterior–ventral (AV)	7	33	10		2.10	1.56	1.89	18.85
anterior–nasall (AN)	7	29	14			0.02	0.00	8.98
anterior–dorsal (AD)	7	26	12				0.02	9.49
anterior–temporal (AT)	7	25	12					8.62
peripheral (P)	8	28	24					

Fig. S12

stage	anterior		peripheral		p-value (t-test)
	lens	cell	lens	cell	
26 hpf	7	49	7	49	0.220
34 hpf	6	42	8	56	0.018
49 hpf	7	49	8	56	0.011
58 hpf	7	49	8	56	0.410
77 hpf	6	41	5	34	0.017

Fig. S13

stage	anterior		peripheral		p value (t-test)
	lens	cell	lens	cell	
26 hpf	6	47	6	45	0.820
34 hpf	6	51	6	48	0.098
49 hpf	7	52	6	38	0.046
58 hpf	6	47	6	34	0.001
77 hpf	5	39	6	25	0.002

Fig. S14

stage	anterior		peripheral		p value (t-test)
	lens	cell	lens	cell	
26 hpf	8	56	7	49	0.360
34 hpf	6	42	8	56	0.950
49 hpf	7	48	8	55	3.23x10-6
58 hpf	7	49	8	56	3.03x10-13
77 hpf	6	42	5	35	2.86x10-10

Fig. S18

stage	WT	hab	p value (t-test)
	slice	slice	
50 hpf	7	8	0.130
72 hpf	9	10	0.240

Fig. S19

stage	region	wild-type sibling		hab		p-value (t-test)
		lens	cell	lens	cell	
32 hpf	anterior	8	19	6	14	0.995
	peripheral	8	22	7	22	0.397
50 hpf	anterior	6	13	6	19	0.435
	peripheral	7	19	6	24	0.041

Fig. S6. Number of samples and statistical analyses. Number of samples and statistical analyses for Fig. 2B–D, Fig. 3A, Fig. 4B, Fig. 5E, Fig. 6A,D, Fig. 7B–D,F and supplementary material Figs S9, S11–S14, S18 and S19. Statistical significant p-value ($p<0.05$) is indicated by blue color.

Biology Open (2014) 000, 1–13 doi:10.1242/bio.20149563

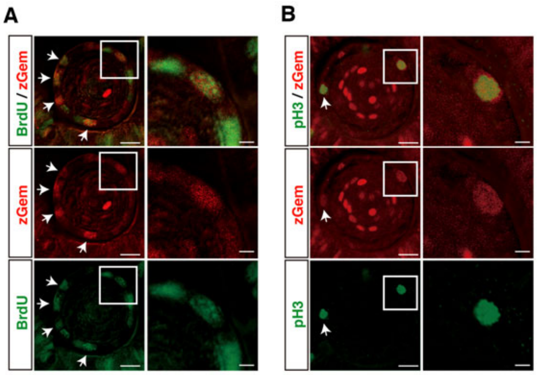

Fig. S7. mCherry-zGem is expressed during S and M phase.
(A) Labeling of the *Tg(EF1α:mCherry-zGem)* lens with anti-BrdU antibody. Arrows indicate mCherry-zGem-positive cells (red) that also incorporate BrdU (green). Right panels are higher magnification versions of the squares in the left panels. (B) Labeling of the *Tg(EF1α:mCherry-zGem)* lens with anti-pH3 antibody. The arrow indicates an mCherry-zGem-positive cell (red), labeled with pH3 (green). Right panels are higher magnification versions of the squares in the left panels. Scale bars: 20 µm (left panels) and 2 µm (right panels).

Biology Open

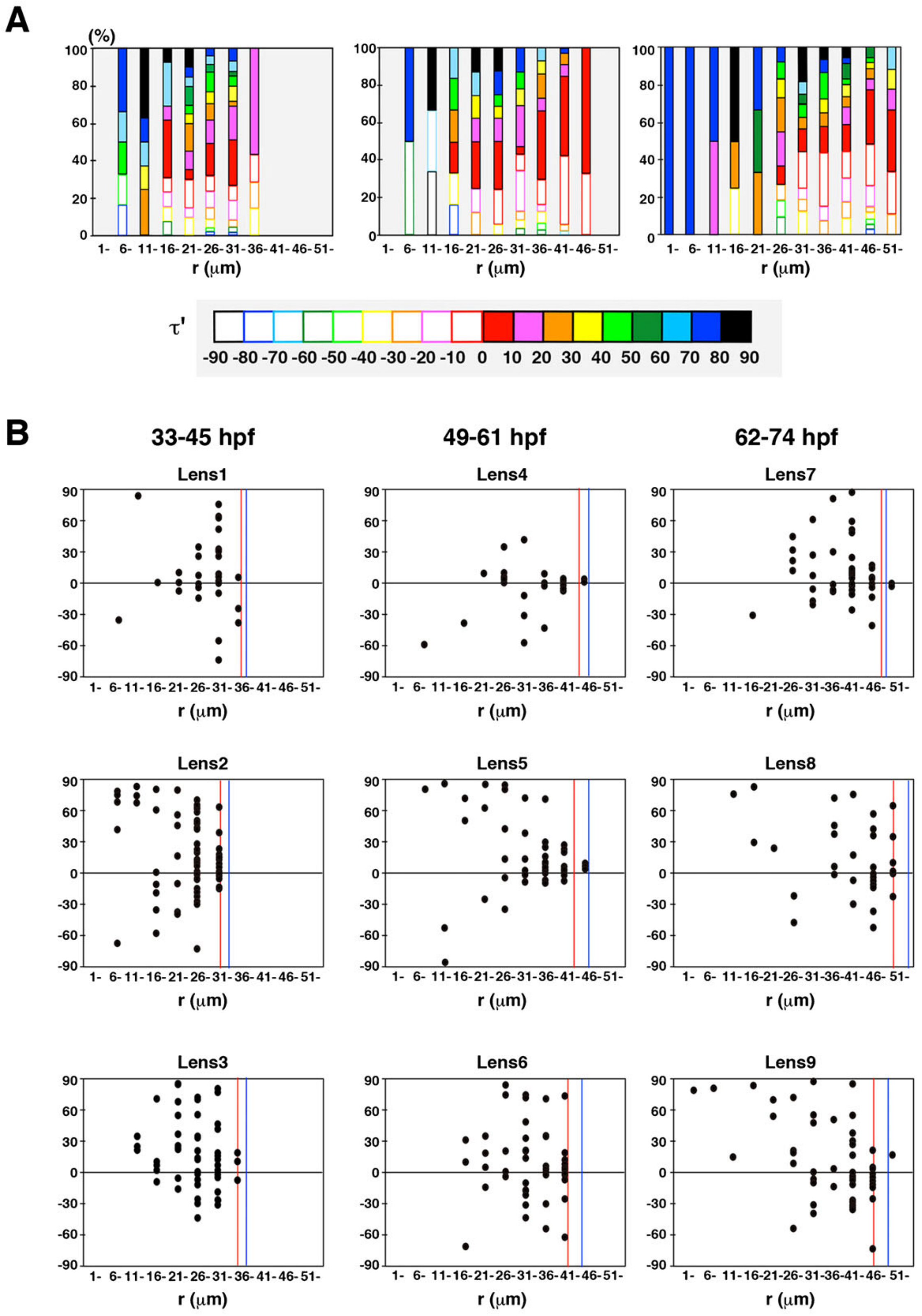

Fig. S8. Cell-division orientation of individual lens epithelial cells. (A) Histogram of cell-division orientation along the r-axis. Closed and open squares indicate clockwise and counter-clockwise cell-division orientation, respectively. The clockwise orientation seems to be more prominent than the counter-clockwise orientation. (B) Plotting of τ' values of individual mitotic cells along the r-axis of nine wild-type lenses shown in supplementary material Fig. S1. The pattern is variable, especially in the anterior region, depending on individual lenses. Red and blue lines indicate equatorial positions at the beginning and end of the scanned period, respectively.

Biology Open

Biology Open (2014) 000, 1–13 doi:10.1242/bio.20149563

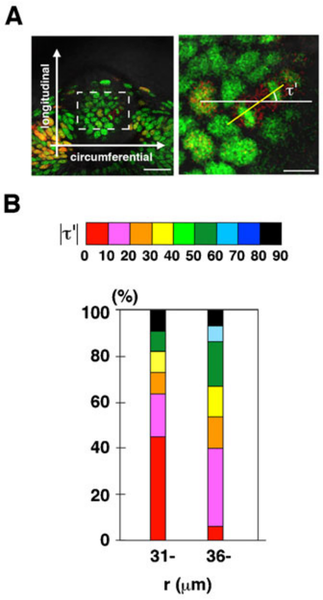

Fig. S9. Measurement of cell-division orientation using time-lapse imaging data acquired from a ventral view. (A) Ventral view image of equatorial region of *Tg(h2afv:GFP; EF1α:mCherry-zGem)* lens. The right panel displays a higher magnification view of the square shown in the left panel. The angle τ′ was directly measured as the angle between the line that connects two daughter nuclei (yellow line) and a horizontal line.
(B) Histogram of the percentage of absolute τ′ angle in two peripheral zones for 31–35 μm and 36–40 μm. Cell division with circumferential orientation (τ′<40) is around 80% in 31–35 μm and 60% in 36–40 μm, demonstrating that circumferential cell division is very active. Scale bars: 20 μm (A, left panel) and 5 μm (A, right panel).

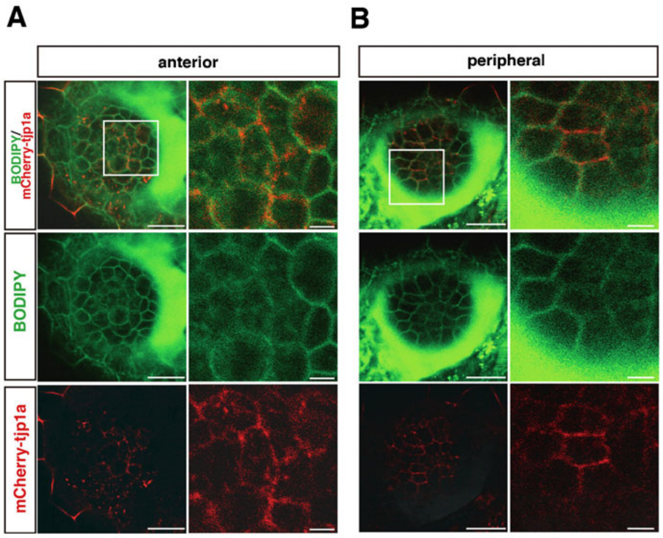

Fig. S10. Fluorescent signal of Bodipy ceramide overlaps with that of mCherry-tjp1. Fluorescent signals of mCherry-tjp1a (red) and Bodipy ceramide (green) were scanned in anterior (A) and peripheral (B) regions at 48 hpf. Middle and bottom panels are green and red channels. Right panels indicate high-magnification images of squares in left panels. Scale bars: 20 μm (left panels) and 5 μm (right panels).

Biology Open

Biology Open (2014) 000, 1–13 doi:10.1242/bio.20149563

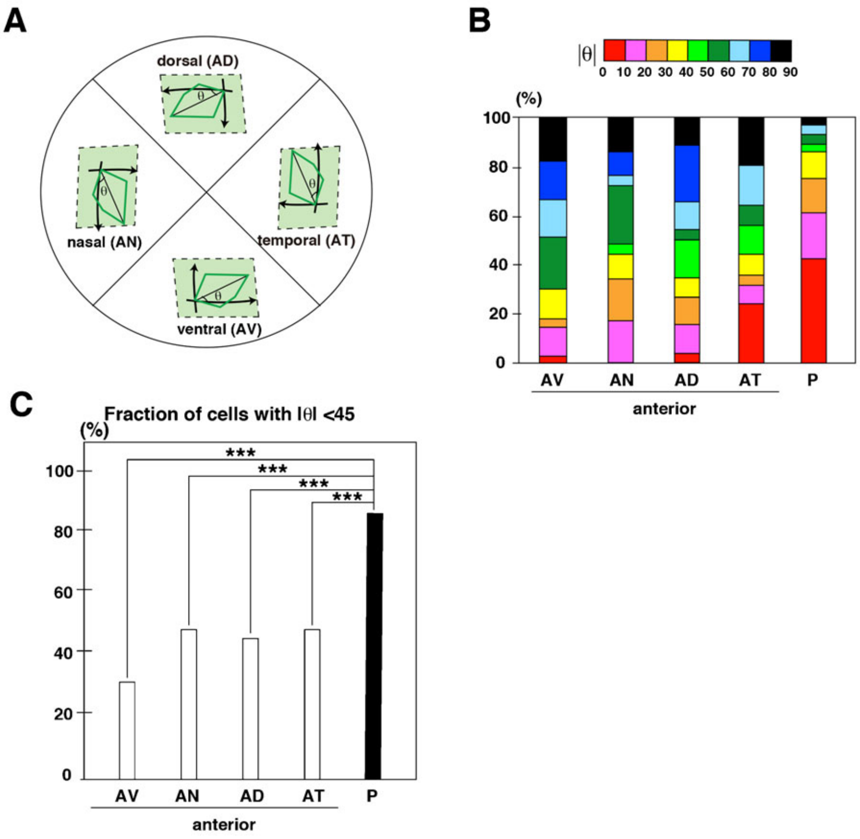

Fig. S11. Long-axis orientation in subdomains of the anterior region. (A) The anterior region is separated into four subdomains: nasal (AN), temporal (AT), ventral (AV), and dorsal (AD) regions. Longitudinal and circumferential axes are defined. (B) The percentage of long-axis orientation (absolute θ value) in nasal (AN), temporal (AT), ventral (AV), and dorsal (AD) subdomains, and the peripheral region. The percentage of orientation with $\theta<40$ is lower in all anterior subdomains than in the peripheral region. (C) Fraction of cells with $\theta<45$ in anterior subdomains and peripheral region. Probabilities were calculated using a χ^2-test: ***$p<0.005$.

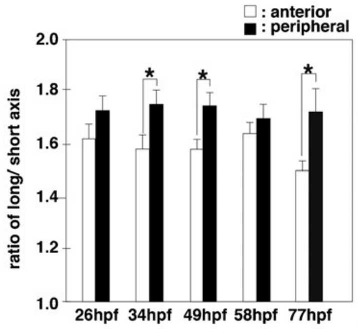

Fig. S12. Ratio of the long axis to the short axis of lens epithelial cells. Long and short axes were measured as Feret's maximum and minimum diameters using Image J software. The ratio is higher in the peripheral region than in the anterior region, and the difference was significant at 34, 49 and 77 hpf (*$p<0.05$, t-test). Bars and lines indicate average and standard error.

Biology Open

Biology Open (2014) 000, 1–13 doi:10.1242/bio.20149563

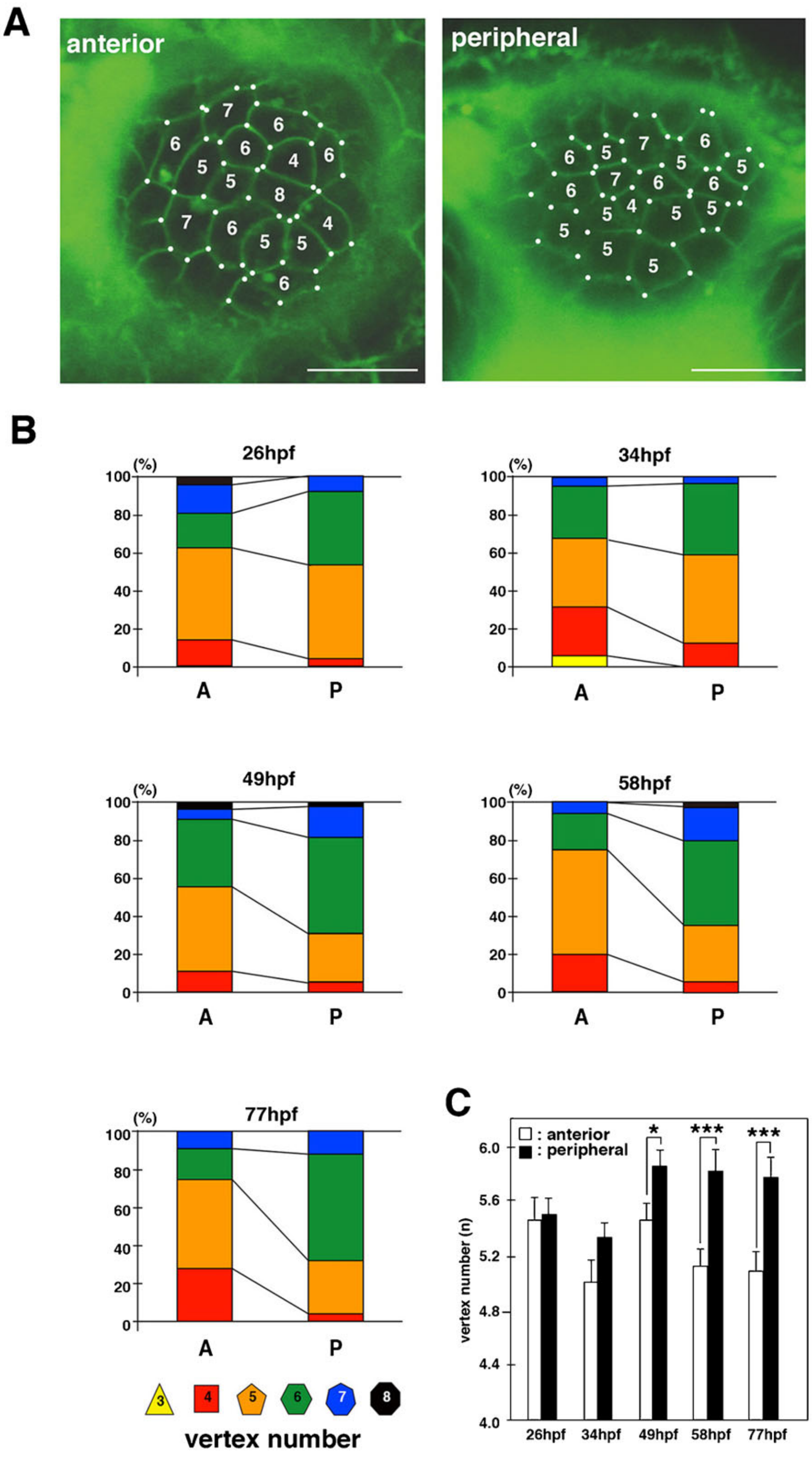

Fig. S13. Vertex number of lens epithelial cells in anterior and peripheral regions. (A) Anterior and peripheral regions in 49 hpf zebrafish lenses labeled with Bodipy ceramide. Vertices are indicated by dots and their numbers are assigned to individual epithelial cells. (B) Percentage of the vertex number of lens epithelial cells in anterior and peripheral regions at 26, 34, 49, 58 and 77 hpf. (C) Average vertex numbers in anterior and peripheral regions. Bars and lines indicate averages and standard errors. Probabilities were calculated by t-test: *$p<0.05$, ***$p<0.005$. Scale bars: 20 µm.

Biology Open (2014) 000, 1–13 doi:10.1242/bio.20149563

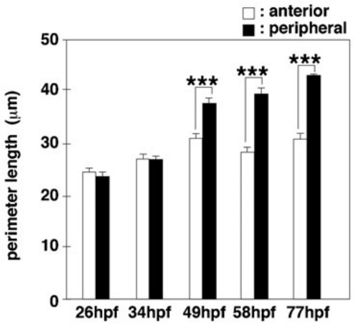

Fig. S14. Perimeter lengths of lens epithelial cells. Perimeter lengths of individual cells in the anterior and peripheral regions. Perimeter lengths did not differ between anterior and peripheral regions at 26 and 34 hpf, but were significantly higher in the peripheral region than in the anterior region after 49 hpf (***$p < 0.005$, t-test). Bars and lines indicate average and standard error.

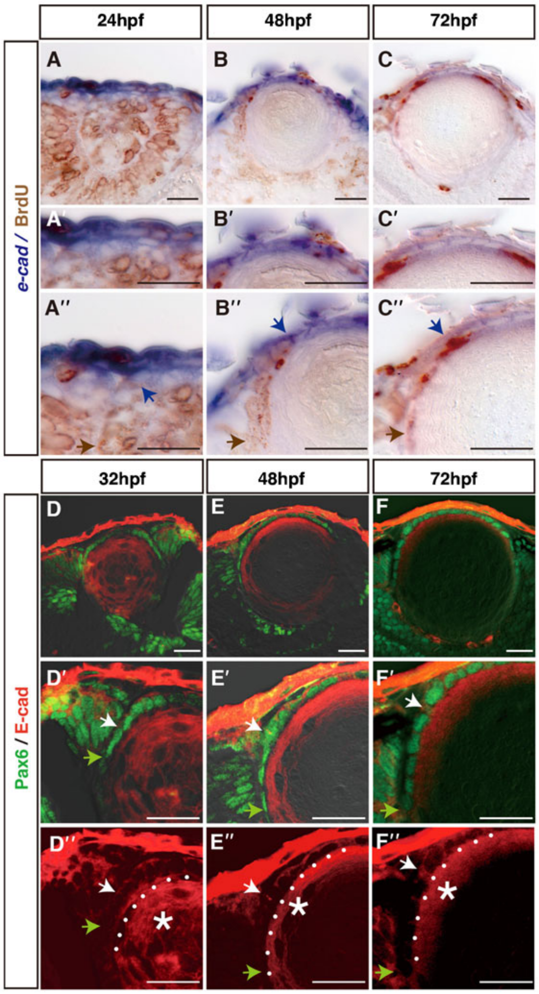

Fig. S15. Expression of E-cadherin mRNA and protein in the lens. (A–C) *e-cadherin* mRNA expression (blue) and BrdU incorporation (brown) in lenses at 24 hpf (A–A″), 48 hpf (B–B″), and 72 hpf (C–C″). (A′–C′) and (A″–C″) indicate high magnification of anterior and peripheral regions of (A–C), respectively. Blue and brown arrows indicate the posterior border of *e-cadherin* mRNA expression and BrdU-incorporated cells, respectively, in lens epithelium. (D–F) Labeling of zebrafish lens with anti-E-cadherin (red) and anti-Pax6 (green) antibodies at 32 hpf (D–D″), 48 hpf (E–E″), and 72 hpf (F–F″). (D′–F′) indicate high magnification of the peripheral region of (D–F). (D″–F″) is the red channel of (D′–F′). White dotted lines indicate the interface between lens epithelium and lens fibers. Anti-E-cadherin antibody recognizes not only lens epithelial cells, but also early differentiating lens fibers (asterisks in D″–F″). Lens fiber cells do not express *e-cadherin* mRNA (A–C). It is likely that the lens fiber signals are due to non-specific staining, because lens epithelial signals disappear, but these lens fiber signals continued to be detected in zebrafish *e-cadherin* mutants (supplementary material Fig. S16C,D). White and green arrows indicate the posterior borders of E-cadherin and Pax6 protein expression, respectively, in the lens epithelium. Scale bars: 20 μm.

Biology Open

 Biology Open (2014) 000, 1–13 doi:10.1242/bio.20149563

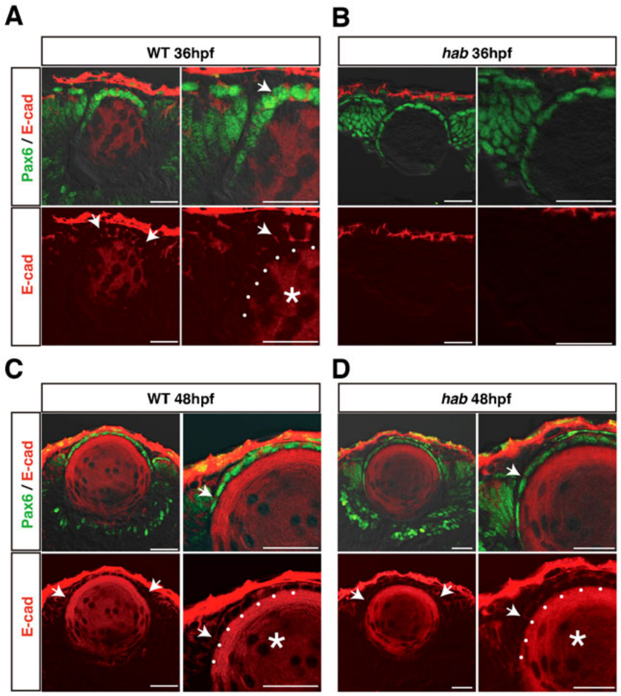

Fig. S16. E-cadherin expression is reduced in *hab*^{rk3} mutant lenses. (A,B) Labeling of 36 hpf wild-type sibling (A) and *hab*^{rk3} mutant (B) lenses with anti-E-cadherin (red) and anti-Pax6 (green) antibodies. Bottom panels are red channels. E-cadherin expression was detected in anterior lens epithelium in wild-type siblings (A), but not in *hab*^{rk3} mutants (B). (C,D) Labeling of 48 hpf wild-type sibling (C) and *hab*^{rk3} mutant (D) lenses with anti-E-cadherin (red) and anti-Pax6 (green) antibodies. Bottom panels are red channels. White dotted lines indicate the interface between lens epithelium and lens fibers. Anti-E-cadherin antibody detects signals in lens epithelial cells and lens fibers (asterisks), the latter of which does not express *e-cadherin* mRNA. E-cadherin expression was detected in anterior lens epithelium in wild-type siblings (C), but reduced in the *hab*^{rk3} mutants (D). Since antibody signals in lens fiber cells are detected in *hab*^{rk3} mutants, these lens fiber signals are likely due to non-specific staining. Arrows indicate the posterior border of E-cadherin expression. Scale bars: 20 µm.

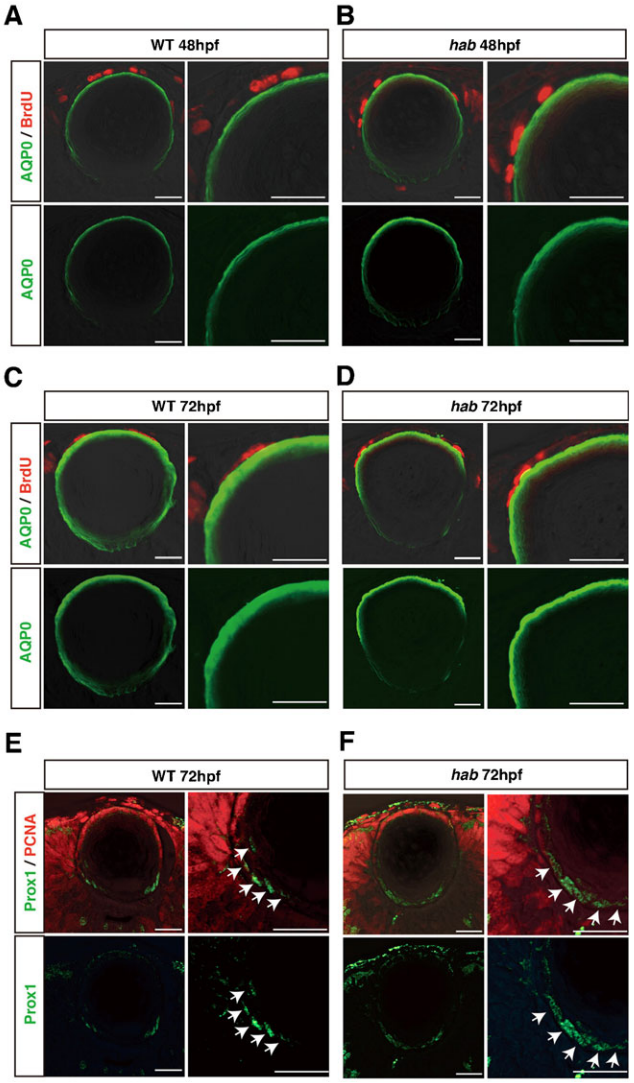

Fig. S17. Lens fiber differentiation normally proceeds in *hab*^{rk3} mutants. (A–D) Labeling of wild-type (A,C) and *hab*^{rk3} mutant (B,D) lenses with anti-AQP0 antibody (green) and BrdU incorporation (red) at 48 and 72 hpf. Bottom panels are green channels. AQP0 is expressed in differentiated fiber cells underneath lens epithelium, in which some epithelial cells are BrdU-positive, in wild type and and *hab*^{rk3} mutants. (E,F) Labeling of wild-type (E) and *hab*^{rk3} mutant (F) lenses with anti-Prox1 antibody (green) and anti-PCNA antibody (red) at 72 hpf. Bottom panels are green channels. Prox1 expression (arrowheads) was detected in wild type and *hab*^{rk3} mutants. Scale bars: 20 µm.

Biology Open

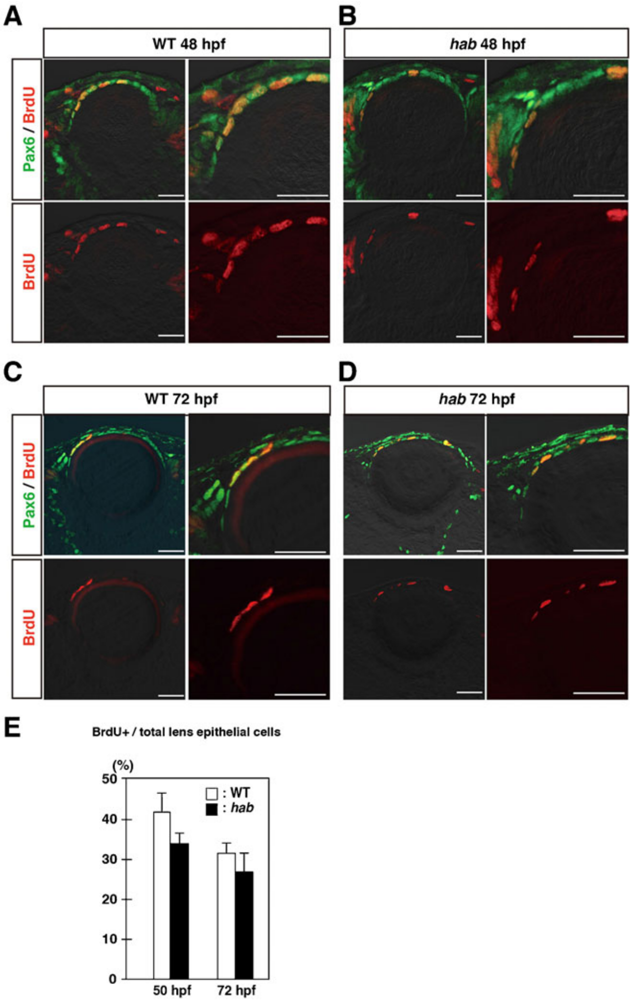

Fig. S18. BrdU incorporation seems to be normal in *hab*^{rk3} mutants. (A–D) Labeling of wild-type (A,C) and the *hab*^{rk3} mutant (B,D) lenses with anti-Pax6 antibody (green) and BrdU incorporation (red) at 48 and 72 hpf. Bottom panels are red channels. BrdU incorporation was observed in Pax6-positive lens epithelium in *hab*^{rk3} mutants. (E) The percentage of BrdU-positive cells relative to Pax6 positive cells in the lens epithelium. The percentage of BrdU-positive cells was slightly lower in *hab*^{rk3} mutants than in wild type, but the difference was not significant. Bars and lines indicate average and standard error. Scale bars: 20 µm.

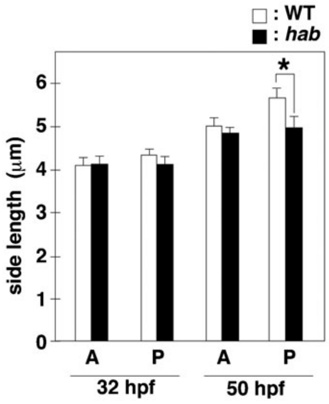

Fig. S19. Side length of hexagonal lens epithelial cells in wild type and *hab*^{rk3} mutants. Histogram of side length of lens epithelial cells in the anterior and peripheral regions of wild type and *hab*^{rk3} mutant lenses at 32 hpf and 50 hpf. Side length of peripheral epithelial cells is shorter in *hab*^{rk3} mutants at 32 hpf. Side length of both anterior and peripheral epithelial cells is shorter in *hab*^{rk3} mutants at 50 hpf. In particular, the difference between wild type and *hab*^{rk3} mutants is significant in the peripheral region at 50 hpf (*$p<0.05$, t-test). Bars and lines indicate means and standard errors.

Table S1. Antibodies

Antibody	Dilution	Company/Clone
Cdh1 (E-cadherin)	1:100	BD Transduction Laboratories; 610181
Pax6	1:200	Covance; PRB-278P
Prox1	1:200	Chemicon; AB5475
Aquaporin 0 (AQP0)	1:500	Millipore; AB3071
PCNA	1:100	Sigma; P8825 (PC-10)
BrdU	1:200	Serotec; MCA2060T
Phosphorylated Histone H3	1:200	Santa Cruz; SC-8656
Rat HRP-conjugate IgG	1:500	Santa Cruz; SC-2006

Biology Open (2014) 000, 1–13 doi:10.1242/bio.20149563

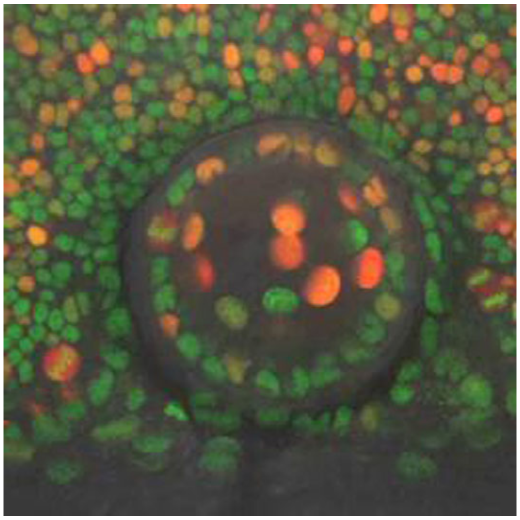

Movie 1. 3D scanning of 37 hpf wild type lenses combined with zebrafish transgenic line *Tg(h2afv:GFP; EF1α:mCherry-zGem)*.

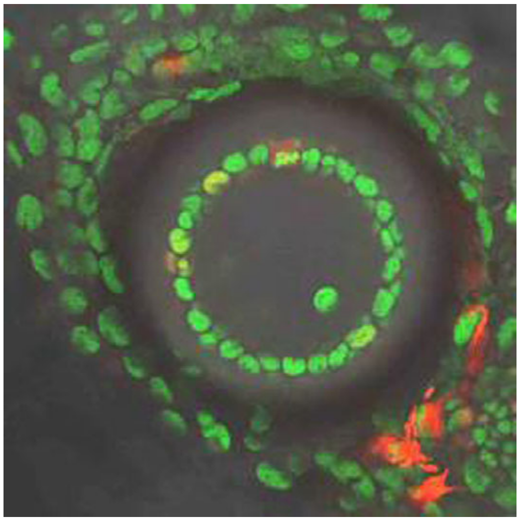

Movie 2. 3D scanning of 49 hpf wild type lenses combined with zebrafish transgenic line *Tg(h2afv:GFP; EF1α:mCherry-zGem)*.

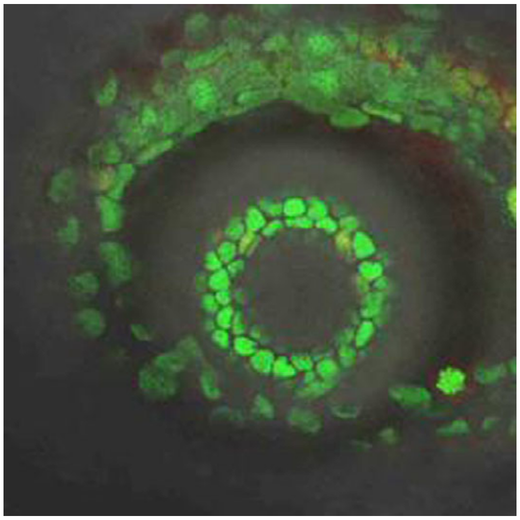

Movie 3. 3D scanning of 62 hpf wild type lenses combined with zebrafish transgenic line *Tg(h2afv:GFP; EF1α:mCherry-zGem)*.

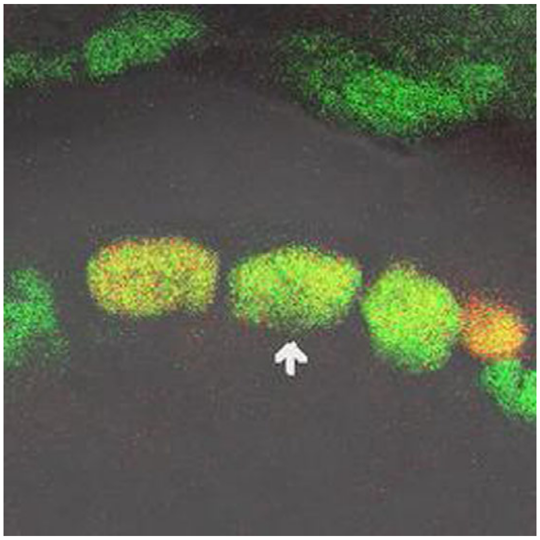

Movie 4. Cell-cycle progression of lens epithelial cells from S to M phases in zebrafish transgenic line *Tg(h2afv:GFP; EF1α:mCherry-zGem)*.

Biology Open (2014) 000, 1–13 doi:10.1242/bio.20149563

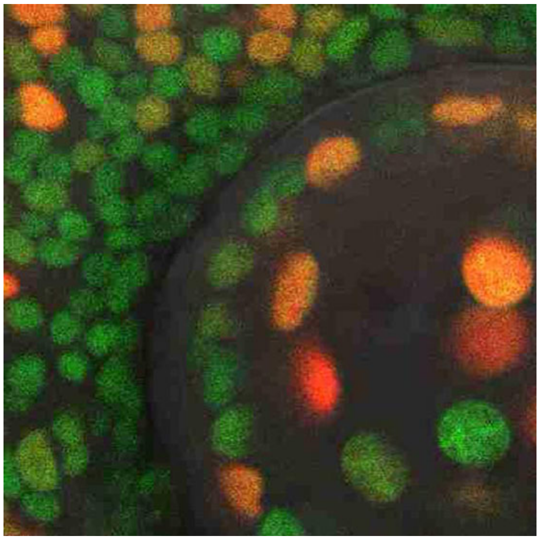

Movie 5. Mitoses of wild type lens epithelial cells in zebrafish transgenic line *Tg(h2afv:GFP; EF1α:mCherry-zGem)*.

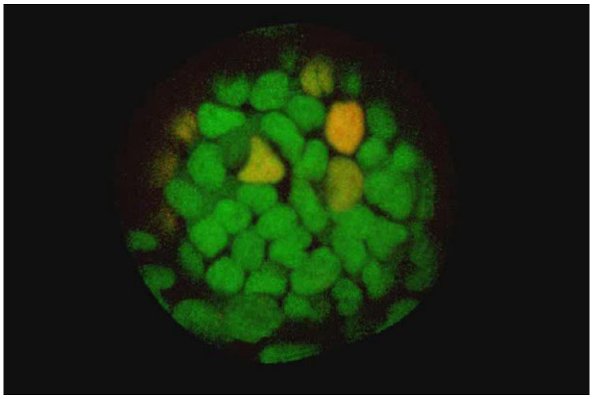

Movie 6. Time-lapse movie of anterior region of wild-type lens combined with zebrafish transgenic line *Tg(h2afv:GFP; EF1α:mCherry-zGem)* from 33 to 45 hpf.

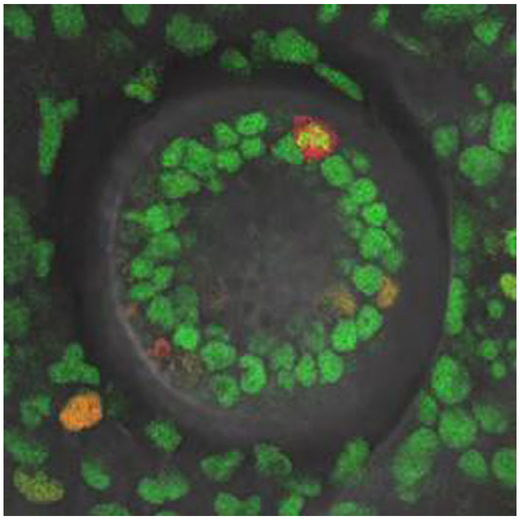

Movie 7. 3D scanning of *hab*^{rk3} mutant lenses combined with zebrafish transgenic line *Tg(h2afv:GFP; EF1α:mCherry-zGem)* at 33 hpf.

Biology Open
